# Assessment of Ten Insulin Resistance Surrogate Indexes Predicts New-Onset Cardiovascular Disease Incidence in Patients with Prediabetes or Diabetes: Insights from CHARLS Data with Machine Learning Analysis

**DOI:** 10.5334/gh.1532

**Published:** 2026-03-12

**Authors:** Hang Xie, Chaoying Yan, Yi Zheng, Haoyu Wu

**Affiliations:** 1 Department of Cardiovascular Medicine, The First Affiliated Hospital of Xi’an Jiaotong University, Xi’an 710061, Shaanxi, China; 2 Department of Anesthesiology, The First Affiliated Hospital of Xi’an Jiaotong University, 710061, Xi’an, Shaanxi, China; 3 Department of Dermatology, The Second Affiliated Hospital of Xi’an Jiaotong University, Xi’an, China

**Keywords:** Insulin resistance, cardiovascular disease, Diabetes mellitus, Machine learning, Risk prediction

## Abstract

**Objective:**

Insulin resistance (IR) is a key driver of prediabetes, type 2 diabetes, and cardiovascular disease (CVD) risk. This study evaluated the predictive performance of ten IR surrogate indexes (TyG, TyG-BMI, TyG-WC, TyG-WHtR, METS-IR, AIP, TyHGB, CTI, eGDR, CVAI) for new-onset CVD in Chinese patients with prediabetes or diabetes, aiming to identify the most effective index for cardiovascular risk stratification.

**Methods:**

This longitudinal cohort study analyzed 3,532 middle-aged and elderly participants from the China Health and Retirement Longitudinal Study (CHARLS) baseline (Wave 1), with incident CVD events assessed at follow-up (Wave 4). Ten IR surrogate indexes were calculated at baseline. Multivariate logistic regression, adjusted for confounders, assessed associations between these indexes and CVD. Non-linear relationships were explored using restricted cubic spline analyses. Nine machine learning algorithms were employed to develop predictive models, with performance evaluated via receiver operating characteristic (ROC) curves, calibration curves, and decision curve analysis.

**Results:**

During follow-up, 874 participants (24.7%) developed CVD. Each standard deviation increase in eGDR was associated with reduced CVD risk (OR = 0.822, 95% CI: 0.696–0.969), while CVAI was linked to increased risk (OR = 1.124, 95% CI: 1.028–1.229). Compared to the lowest quartile, the highest eGDR quartile had a 47.3% lower CVD risk (OR = 0.527, 95% CI: 0.353–0.789, P = 0.0018), and the highest CVAI quartile had a 33.1% higher risk (OR = 1.331, 95% CI: 1.038–1.709, P = 0.0243). Incorporating eGDR and CVAI into machine learning models, particularly K-Nearest Neighbors (KNN), enhanced discrimination (AUC = 0.936, 95% CI: 0.928–0.943).

**Conclusion:**

eGDR and CVAI outperformed other IR indexes in predicting CVD in Chinese patients with prediabetes or diabetes. Their integration into KNN models significantly improved risk stratification, suggesting their utility as accessible clinical tools for early identification and intervention to reduce CVD burden.

## 1. Introduction

Cardiovascular disease (CVD), encompassing heart disease and stroke, remains the leading cause of morbidity and mortality globally (1,2). In 2020, CVD accounted for an age-standardized incidence of 411.8 per 100,000 and a mortality of 245.39 per 100,000 in China (3). Atherosclerosis, the primary pathological driver, underlies clinical manifestations such as myocardial infarction and ischemic stroke (4). Identifying high-risk individuals is essential for effective prevention strategies (5).

Insulin resistance (IR) is a critical pathophysiological mechanism contributing to CVD development and mortality (6,7). Individuals with prediabetes and diabetes face significantly elevated stroke risk, likely related to multiple metabolic abnormalities including IR, hyperglycemia, dyslipidemia, and hypertension (8,9). While the hyperinsulinemic-euglycemic clamp remains the gold standard for assessing IR, its complexity precludes use in large-scale studies, necessitating surrogate markers (10). This has led to the development and use of various surrogate markers for IR (11).

Multiple IR surrogate indexes have been developed using readily available clinical parameters (11,12,13). These include the triglyceride-glucose (TyG) index and its derivatives (TyG-BMI, TyG-WHtR), estimated glucose disposal rate (eGDR), atherogenic index of plasma (AIP), and Chinese Visceral Adiposity Index (CVAI), each reflecting different metabolic abnormalities (11,14,15,16,17). While individual indexes have shown associations with CVD risk (17,18,19,20), comprehensive comparisons of their predictive values in Chinese populations with abnormal glucose metabolism remain lacking. This study evaluates ten IR surrogate indexes for predicting new-onset CVD in patients with prediabetes or diabetes using China Health and Retirement Longitudinal Study data with machine learning analysis.

## 2. Methods

### 2.1 Study design and population

This study utilized data from the China Health and Retirement Longitudinal Study (CHARLS), a nationally representative longitudinal survey designed to examine the health, social, and economic status of Chinese residents aged 45 years and older. The baseline survey (Wave 1) was conducted in 2011, with follow-up surveys conducted every two years. The study design of CHARLS employed a multistage probability sampling strategy covering 150 counties/districts from 28 provinces across China, ensuring broad geographical representation and socioeconomic diversity. For our analysis, we focused on participants from the baseline survey (Wave 1) who had blood samples collected and were followed up until Wave 4. Blood samples in CHARLS were collected by trained nurses following standardized protocols, and all specimens were analyzed at a central laboratory to ensure consistency in testing procedures and quality control. Blood biomarkers measured included fasting blood glucose (FBG), glycated hemoglobin (HbA1c), triglycerides (TG), total cholesterol (TC), high-density lipoprotein cholesterol (HDL-C), low-density lipoprotein cholesterol (LDL-C), creatinine, blood urea nitrogen (BUN), uric acid (UA), and high-sensitivity C-reactive protein (hs-CRP), which were essential for calculating the ten IR surrogate indexes.

The participant selection process is illustrated in Figure S1. From the initial 11,847 participants at baseline with available blood samples, we excluded 1,964 participants with missing demographic data and 1,045 participants without follow-up data until Wave 4. We further excluded 222 participants with incomplete data on HbA1c, TG, HDL, FBG, or CRP, which were essential for calculating the IR indexes. Among the 8,616 participants with complete data on all ten IR surrogate indexes at Wave 1, we applied additional exclusion criteria: 270 participants younger than 45 years old; 1,205 participants with existing heart disease or stroke at baseline; 229 participants with extreme values in any of the ten IR surrogate indexes (defined as values exceeding mean ± 3 standard deviations); 2,866 participants without prediabetes or diabetes; and 514 participants without cardiovascular disease outcome data at follow-up. Ultimately, 3,532 participants with prediabetes or diabetes at baseline were included in the final analysis. All participants provided written informed consent before taking part in the survey. The CHARLS study was approved by the Ethical Review Committee of Peking University (IRB00001052-11015), and our current analysis complied with the principles of the Declaration of Helsinki.

### 2.2 Definition of prediabetes and diabetes

Prediabetes and diabetes were defined according to established clinical criteria based on laboratory measurements and self-reported information using the American Diabetes Association diagnostic criteria (21). Diabetes mellitus was defined by meeting at least one of the following criteria: (1) fasting plasma glucose (FPG) ≥126 mg/dL (7.0 mmol/L), (2) glycated hemoglobin (HbA1c) ≥6.5%, (3) self-reported physician diagnosis of diabetes, or (4) current use of antidiabetic medications. Prediabetes was characterized by an FPG of 100 to 125 mg/dL (5.6 to 6.9 mmol/L) or an HbA1c of 5.7–6.4%, without meeting the criteria for diabetes. All participants classified as having either prediabetes or diabetes at baseline were included in our final analysis to evaluate the predictive performance of IR surrogate indexes for CVD risk in this high-risk population.

### 2.3 Definition of CVD

The primary outcome of our study was incident CVD, defined as the first occurrence of heart disease or stroke during the follow-up period (Wave 4) among participants without a history of CVD at baseline. Heart disease was ascertained through self-reported physician diagnosis based on the standardized question, ‘Has a doctor ever told you that you had a heart attack, coronary heart disease, angina, congestive heart failure, or other heart problems?’ Similarly, stroke was determined by a positive response to the question, ‘Has a doctor ever informed you that you had a stroke?’ This definition of CVD is consistent with previously published studies using CHARLS data (22,23). To ensure data integrity, participants who reported CVD diagnoses at follow-up were required to reaffirm their condition in subsequent waves. In cases where participants denied previously self-reported diagnoses of heart disease or stroke, these inconsistencies were rectified retrospectively by carefully reviewing their medical history across all available waves.

### 2.4 Calculation of ten insulin resistance surrogate indexes

All participants provided venous blood samples after fasting for at least 8 hours. Fasting venous blood samples were collected by medical professionals, stored at -20°C, and then transported by a cold chain transport company to the Chinese Center for Disease Control and Prevention in Beijing for further analysis. Anthropometric measurements were collected following standardized protocols. Body height and weight were measured by trained professionals using calibrated equipment while participants wore light clothing and no shoes. Waist circumference (WC) was measured at the umbilical level using a flexible measuring tape. For each measurement, three readings were taken, and the average value was recorded as the final measurement to ensure accuracy and reliability. Body mass index (BMI) was calculated as weight in kilograms divided by the square of height in meters (kg/m²). Waist-to-height ratio (WHtR) was calculated by dividing waist circumference by height. The methods for calculating ten IR indices are provided in Supplementary Materials I.

### 2.5 Covariates

Sociodemographic and clinical characteristics were collected at baseline through comprehensive interviews and physical examinations. Sociodemographic variables included age, sex, education level (categorized as elementary school or below, middle school, and college or above), marital status (married and unmarried), residential area (urban or rural), smoking status (never, former, or current smoker), and alcohol consumption (never, former, or current drinker). Hypertension was defined by meeting one of the following criteria: (1) SBP ≥ 140 mmHg, (2) DBP ≥ 90 mmHg, (3) self-reported hypertension diagnosed by a physician, (4) taking antihypertensive medications. Dyslipidemia was identified by TG ≥ 150 mg/dL, TC ≥ 240 mg/dL, HDL-C < 40 mg/dL, LDL-C ≥ 160 mg/dL, current use of lipid-lowering drugs, or self-reported dyslipidemia diagnosed by a physician. Medical history was assessed through self-reported physician diagnoses of chronic conditions, including heart disease, stroke, liver disease, kidney disease, digestive disease, and cancer. Medication use, particularly for diabetes, hypertension, and dyslipidemia, was also documented.

### 2.6 Missing data processing

The degree of missing data within this study is presented in Figure S2, showing the percentage of missing values across all variables. As illustrated, the majority of variables had less than 5% missing data, with several variables such as gender, age, and marital status showing complete or near-complete data. To address missing values (which constituted approximately 3.8% of total data points) and maintain the most comprehensive sample size possible to better represent the actual population characteristics, we performed multiple imputation using the K-Nearest Neighbors (KNN) algorithm (24). The KNN imputation method was selected for its effectiveness in handling mixed categorical and continuous variables while preserving the relationships between variables. All variables in the analysis were included in the imputation model to ensure the highest quality of imputed values.

### 2.7 Machine learning models development

For the development of predictive models for CVD incidence, we employed a systematic feature selection approach followed by comprehensive model training. Initially, feature selection was performed using the least absolute shrinkage and selection operator (LASSO) algorithm (17,25), which effectively performs both variable selection and regularization. This approach improves model interpretability and helps prevent overfitting by shrinking less important variables to zero. Concurrently, we applied the Boruta algorithm (26), a robust ensemble feature selection method demonstrating particular efficacy in high-dimensional data environments with nonlinear relationships, to determine the most critical features associated with incident CVD. Following feature selection, the Synthetic Minority Over-sampling Technique (SMOTE) was applied to achieve a balanced distribution between CVD and non-CVD groups. To enhance model robustness and predictive accuracy, we utilized the intersection of features identified by both LASSO and Boruta algorithms as our final feature set. Using this optimized feature set, we developed and compared nine different machine learning algorithms: KNN, Support Vector Machine (SVM), CatBoost, Gradient Boosting Machine (GBM), XGBoost, LightGBM, Adaptive Boosting (AdaBoost), Neural Network (NN), and Logistic Regression (LR). Hyperparameter optimization was conducted through 10-fold cross-validation using grid search and Bayesian optimization to identify the optimal configuration for each algorithm. Models were evaluated using metrics including area under the receiver operating characteristic curve (AUC), accuracy, precision, recall, sensitivity, specificity, and F1 score. Additionally, calibration curves and decision curve analysis were performed to assess model calibration and clinical utility, respectively.

### 2.8 Statistical analyses

In our statistical analysis approach, we employed a comprehensive framework to evaluate the predictive capabilities of ten IR surrogate indexes for new-onset CVD incidence. For comparing continuous variables across groups, we utilized one-way ANOVA for normally distributed data or Kruskal-Wallis rank sum tests for non-normally distributed variables. Categorical variables were analyzed using Chi-square tests. Data presentation followed standard conventions, with continuous variables expressed as mean (standard deviation, SD) or median (interquartile range, IQR) based on their distribution, while categorical variables were reported as counts (percentages). The ten IR surrogate indexes were analyzed both as continuous variables and as categorical variables stratified by quartiles. To assess the relationship between each IR surrogate index and incident CVD, we constructed four progressively adjusted logistic regression models: Model 1 was unadjusted; Model 2 included adjustments for age and gender; Model 3 incorporated additional adjustments for marital status, smoking status, drinking status, education level, residence, hypertension, dyslipidemia, kidney disease, liver disease, and medication use (antidiabetic drugs, lipid-lowering agents, antihypertensive drugs); and Model 4 further adjusted for TC, LDL, BUN, creatinine, SBP, and DBP. To investigate potential nonlinear relationships between IR surrogate indexes and CVD risk, we performed restricted cubic spline analyses with 4 knots positioned at the 5th, 35th, 65th, and 95th percentiles of each index distribution. Subgroup analyses were conducted to evaluate the consistency of associations across different demographic and clinical characteristics. Interactions between IR surrogate indexes and various factors (age, gender, BMI, smoking status, drinking status, hypertension, and dyslipidemia) were assessed to identify potential effect modifiers. The significance of interaction terms was evaluated within the fully adjusted models.

The discriminative ability of each IR surrogate index for predicting incident CVD was evaluated using receiver operating characteristic (ROC) curve analysis, with area under the curve (AUC) serving as the primary metric. Comparisons between the AUCs of different IR indexes were performed using DeLong’s test to identify the index with superior predictive performance. To quantify the incremental predictive value of adding IR surrogate indexes to baseline risk models, we compared the AUCs of models with and without these indexes using DeLong’s test. Additionally, we calculated other performance metrics, including sensitivity, specificity, accuracy, precision, recall, and F1 score. To assess model calibration and clinical utility, we generated calibration curves and performed decision curve analysis (DCA). Calibration curves plotted the observed versus predicted probabilities to evaluate the agreement between the model’s predictions and actual outcomes. DCA was employed to determine the net benefit of using each IR index for clinical decision-making across a range of threshold probabilities. To enhance model interpretability and understand feature contributions to predictions, we applied SHAP (SHapley Additive exPlanations) analysis to the optimal KNN model.

To enhance the robustness of our findings and assess their stability under different conditions, we conducted several sensitivity analyses: (1) excluding participants with incomplete covariate data; (2) excluding those receiving antihyperglycemic, antihypertensive, or lipid-lowering treatments at baseline; (3) excluding participants who developed CVD in wave 2 during follow-up; and (4) calculating E-values to quantify the minimum strength of association that an unmeasured confounder would need to have with both the exposure and outcome to fully explain away the observed association, thereby assessing the potential impact of unmeasured confounding; (5) conducting stratified analyses by glycemic status to examine whether the associations differed between participants with prediabetes and diabetes. All statistical analyses were conducted using R Statistical Software (Version 4.2.2, http://www.R-project.org, The R Foundation). Machine learning model development and evaluation were performed using the ‘caret’, ‘glmnet’, ‘randomForest’, ‘xgboost’, ‘lightgbm’, ‘catboost’, ‘kernlab’, ‘nnet’, and ‘Boruta’ packages. ROC curve analyses were conducted using the ‘pROC’ package. For all analyses, a two-sided P-value < 0.05 was considered statistically significant.

## 3. Results

### 3.1 Baseline characteristics

A total of 3,532 participants with prediabetes or diabetes were enrolled, of whom 874 (24.7%) developed new-onset CVD during follow-up. Table 1 presents baseline characteristics stratified by CVD status. Participants who developed CVD were more likely to be female (59.8% vs. 52.9%, *P* < 0.001), older (60.74 ± 8.59 vs. 58.32 ± 8.55 years, *P* < 0.001), have a higher BMI (24.48 ± 3.99 vs. 23.63 ± 3.70 kg/m², *P* < 0.001), have a higher blood pressure (SBP: 134.15 ± 21.56 vs. 129.08 ± 20.01 mmHg, *P* < 0.001), and have a higher prevalence of hypertension (54.9% vs. 38.0%, *P* < 0.001) and dyslipidemia (17.3% vs. 6.5%, *P* < 0.001). All ten IR surrogate indexes differed significantly between groups. Participants with incident CVD had lower eGDR (8.38 ± 2.34 vs. 9.24 ± 2.20, *P* < 0.001) and higher CVAI (109.43 ± 40.66 vs. 96.63 ± 38.68, *P* < 0.001), TyG (8.90 ± 0.65 vs. 8.82 ± 0.63, *P* = 0.002), and other IR indices (all *P* < 0.05), indicating greater IR was associated with increased CVD risk.

**Table 1:** Baseline characteristics of the study population according to CVD.


VARIABLES	WITHOUT CVD (N = 2658)	WITH CVD (N = 874)	*p*

HbA1c (mean, SD)	5.39 (0.89)	5.48 (0.95)	0.017

FBG (mean, SD)	120.24 (37.68)	122.88 (41.46)	0.08

UA (mean, SD)	4.47 (1.24)	4.41 (1.27)	0.196

Creatinine (mean, SD)	0.78 (0.19)	0.77 (0.20)	0.686

TG (mean, SD)	136.78 (88.28)	143.45 (86.23)	0.051

TC (mean, SD)	197.70 (38.98)	199.52 (38.78)	0.229

HDL (mean, SD)	51.31 (15.40)	49.84 (15.15)	0.014

CRP (mean, SD)	2.70 (7.69)	2.93 (7.67)	0.438

WC (mean, SD)	85.02 (11.15)	87.66 (11.90)	<0.001

Weight (mean, SD)	59.01 (10.97)	60.74 (12.06)	<0.001

Height (mean, SD)	1.58 (0.08)	1.57 (0.09)	0.072

BUN (mean, SD)	16.00 (4.56)	15.64 (4.42)	0.041

LDL (mean, SD)	119.24 (34.92)	120.93 (36.74)	0.22

Gender (%)			

Female	1407 (52.9)	523 (59.8)	<0.001

Male	1251 (47.1)	351 (40.2)	

Age (mean, SD)	58.32 (8.55)	60.74 (8.59)	<0.001

BMI (mean, SD)	23.63 (3.70)	24.48 (3.99)	<0.001

SBP (mean, SD)	129.08 (20.01)	134.15 (21.56)	<0.001

DBP (mean, SD)	75.21 (11.39)	77.26 (12.29)	<0.001

Marital status (%)			

Unmarried	366 (13.8)	152 (17.4)	0.01

Married	2292 (86.2)	722 (82.6)	

Smoking status (%)			

never smoker	1640 (61.7)	571 (65.3)	<0.001

former smoker	201 (7.6)	96 (11.0)	

current smoker	817 (30.7)	207 (23.7)	

Drinking status (%)			

never drinker	1590 (59.8)	544 (62.2)	0.01

former drinker	208 (7.8)	88 (10.1)	

current drinker	860 (32.4)	242 (27.7)	

Education level (%)			

elementary school or below	1891 (71.1)	624 (71.4)	0.049

middle school	708 (26.6)	218 (24.9)	

college or above	59 (2.2)	32 (3.7)	

Rural (%)			

No	398 (15.0)	168 (19.2)	0.004

Yes	2260 (85.0)	706 (80.8)	

Hypertension (%)			

No	1647 (62.0)	394 (45.1)	<0.001

Yes	1011 (38.0)	480 (54.9)	

Dyslipidemia (%)			

No	2485 (93.5)	723 (82.7)	<0.001

Yes	173 (6.5)	151 (17.3)	

Kidney disease (%)			

No	2548 (95.9)	822 (94.1)	0.033

Yes	110 (4.1)	52 (5.9)	

Liver disease (%)			

No	2589 (97.4)	843 (96.5)	0.176

Yes	69 (2.6)	31 (3.5)	

Antidiabetic drugs (%)			

No	2549 (95.9)	804 (92.0)	<0.001

Yes	109 (4.1)	70 (8.0)	

Lipid-lowering agents (%)			

No	2570 (96.7)	800 (91.5)	<0.001

Yes	88 (3.3)	74 (8.5)	

Antihypertensive drugs (%)			

No	2263 (85.1)	604 (69.1)	<0.001

Yes	395 (14.9)	270 (30.9)	

eGDR (mean, SD)	9.24 (2.20)	8.38 (2.34)	<0.001

CVAI (mean, SD)	96.63 (38.68)	109.43 (40.66)	<0.001

TyG (mean, SD)	8.82 (0.63)	8.90 (0.65)	0.002

TyG-BMI (mean, SD)	209.09 (39.27)	218.42 (42.11)	<0.001

TyG-WC (mean, SD)	7.52 (1.24)	7.81 (1.31)	<0.001

TyG-WHtR (mean, SD)	4.77 (0.80)	4.98 (0.84)	<0.001

METS-IR (mean, SD)	36.19 (7.75)	37.99 (8.36)	<0.001

AIP (mean, SD)	0.86 (0.76)	0.95 (0.75)	0.002

TyHGB (mean, SD)	10.23 (3.49)	10.69 (3.80)	0.001

CTI (mean, SD)	8.89 (0.82)	9.00 (0.83)	0.001


### 3.2 Predictive value of ten IR indices for the incidence of CVD

**Figure 1:**
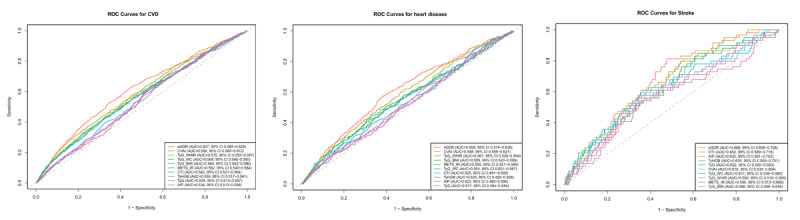
Receiver operating characteristic (ROC) curves comparing the predictive performance of ten insulin resistance surrogate indices for cardiovascular events. **(A)** ROC curves for predicting total CVD events. **(B)** ROC curves for predicting heart disease. **(C)** ROC curves for predicting stroke. Among all ten insulin resistance surrogate indices evaluated, eGDR consistently showed the highest discriminative ability across all cardiovascular outcomes, while CVAI demonstrated the second-best performance for total CVD and heart disease prediction.

To evaluate the discriminative ability of insulin resistance surrogate indices for predicting incident CVD, we performed ROC curve analyses for all ten IR indices (Figure 1). Among these indices, eGDR demonstrated the strongest predictive capability for CVD with the highest AUC value of 0.633 (95% CI: 0.613–0.653), followed by CVAI with an AUC of 0.623 (95% CI: 0.602–0.643). When specifically examining heart disease as an outcome, eGDR again exhibited superior discrimination with an AUC of 0.627 (95% CI: 0.605–0.649), while CVAI showed the second-best performance with an AUC of 0.622 (95% CI: 0.600–0.644). Similarly, for stroke prediction, eGDR maintained its superior predictive performance with an AUC of 0.640 (95% CI: 0.612–0.668), significantly outperforming other indices.

### 3.3 Associations of ten IR indices with new-onset CVD

We examined the associations between ten IR surrogate indices and incident CVD events using progressively adjusted logistic regression models (Table 2). Among all indices evaluated, eGDR and CVAI demonstrated the strongest and most consistent association with CVD risk. The baseline characteristics stratified by quartiles of CVAI and eGDR are presented in Tables S1 and S2, respectively. In the fully adjusted model (Model IV), each standard deviation increase in eGDR was associated with a significant 17.8% reduction in CVD risk (OR = 0.822, 95% CI: 0.696–0.969, *P* = 0.021). When analyzed by quartiles, participants in the highest eGDR quartile (Q4) exhibited a 47.3% lower risk of incident CVD compared to those in the lowest quartile (OR = 0.527, 95% CI: 0.353–0.789, *P* = 0.0018), with a significant dose-response relationship (*P* for trend = 0.0064). Similarly, CVAI showed a positive association with incident CVD. Each standard deviation increase in CVAI was associated with a 12.4% higher risk of CVD in the fully adjusted model (OR = 1.124, 95% CI: 1.028–1.229, *P* = 0.0099). Participants in the highest CVAI quartile had a 33.1% higher risk of developing CVD compared to those in the lowest quartile (OR = 1.331, 95% CI: 1.038–1.709, *P* = 0.0243), with a significant linear trend across quartiles (*P* for trend = 0.0483). While other IR indices (TyG-BMI, TyG-WC, METS-IR, TyG-WHtR) initially showed associations with CVD risk after adjustment for age and gender, these relationships attenuated and became non-significant in the fully adjusted models, suggesting that eGDR and CVAI may be superior predictors of CVD risk in this population.

**Table 2:** Associations of ten insulin resistance surrogate indexes with new-onset cardiovascular disease incidence in patients with prediabetes or diabetes.


	MODEL I OR (95%CI)	P-VALUE	MODEL II OR (95%CI)	P-VALUE	MODEL III OR (95%CI)	P-VALUE	MODEL IV OR (95%CI)	P-VALUE

eGDR (per 1 SD)	0.682(0.631–0.737)	<0.001	0.705(0.651–0.762)	<0.001	0.809(0.686–0.952)	0.0112	0.822(0.696–0.969)	0.021

eGDR quartile								

Q1	1(Reference)		1(Reference)		1(Reference)		1(Reference)	

Q2	0.636(0.519–0.779)	<0.001	0.646(0.526–0.792)	<0.001	0.767(0.604–0.972)	0.0289	0.776(0.610–0.985)	0.0378

Q3	0.387(0.310–0.482)	<0.001	0.426(0.340–0.532)	<0.001	0.502(0.338–0.747)	0.0006	0.519(0.349–0.776)	0.0013

Q4	0.389(0.312–0.484)	<0.001	0.418(0.335–0.521)	<0.001	0.505(0.339–0.753)	0.0007	0.527(0.353–0.789)	0.0018

P for trend		<0.001		<0.001		0.0031		0.0064

CVAI (per 1 SD)	1.383(1.281–1.495)	<0.001	1.324(1.223–1.435)	<0.001	1.137(1.042–1.241)	0.0038	1.124(1.028–1.229)	0.0099

CVAI quartile								

Q1	1(Reference)		1(Reference)		1(Reference)		1(Reference)	

Q2	1.33(1.057–1.684)	0.0155	1.225(0.968–1.552)	0.0915	1.157(0.910–1.472)	0.234	1.134(0.891–1.444)	0.3062

Q3	1.465(1.165–1.847)	0.002	1.298(1.026–1.643)	0.0296	1.086(0.851–1.387)	0.509	1.042(0.815–1.335)	0.7415

Q4	2.343(1.881–2.927)	<0.001	2.047(1.635–2.569)	<0.001	1.383(1.083–1.769)	0.0095	1.331(1.038–1.709)	0.0243

P for trend		<0.001		<0.001		0.0196		0.0483

TyHGB (per 1 SD)	1.130(1.050–1.215)	0.0009	1.153(1.070–1.240)	0.0002	1.044(0.962–1.130)	0.2981	1.049(0.955–1.151)	0.3169

TyHGB quartile								

Q1	1(Reference)		1(Reference)		1(Reference)		1(Reference)	

Q2	1.324(1.061–1.653)	0.0133	1.330(1.064–1.666)	0.0126	1.234(0.982–1.553)	0.0721	1.203(0.955–1.518)	0.1171

Q3	1.231(0.984–1.541)	0.0686	1.261(1.005–1.583)	0.0452	1.065(0.842–1.347)	0.5993	1.027(0.810–1.304)	0.8214

Q4	1.502(1.207–1.872)	0.0003	1.556(1.247–1.945)	<0.001	1.144(0.903–1.449)	0.2658	1.100(0.859–1.410)	0.4484

P for trend		0.0012		0.0004		0.529		0.753

TyG (per 1 SD)	1.125(1.043–1.213)	0.0023	1.131(1.046–1.222)	0.00189	1.031(0.949–1.119)	0.4689	1.029(0.933–1.134)	0.5672

TyG quartile								

Q1	1(Reference)		1(Reference)		1(Reference)		1(Reference)	

Q2	1.228(0.983–1.534)	0.0706	1.215(0.971–1.522)	0.0888	1.130(0.898–1.422)	0.2983	1.107(0.878–1.396)	0.389

Q3	1.303(1.046–1.626)	0.0187	1.269(1.014–1.588)	0.0375	1.114(0.885–1.404)	0.3576	1.085(0.858–1.373)	0.4956

Q4	1.381(1.110–1.721)	0.0039	1.397(1.119–1.746)	0.0033	1.091(0.864–1.378)	0.4627	1.053(0.812–1.364)	0.6987

P for trend		0.0038		0.00378		0.5271		0.7182

TyG-BMI (per 1 SD)	1.254(1.163–1.352)	<0.001	1.305(1.208–1.411)	<0.001	1.125(1.033–1.225)	0.0067	1.119(1.024–1.222)	0.0127

TyG-BMI quartile								

Q1	1(Reference)		1(Reference)		1(Reference)		1(Reference)	

Q2	1.021(0.812–1.283)	0.861	1.100(0.872–1.389)	0.4195	1.009(0.795–1.279)	0.9434	1.002(0.789–1.273)	0.9849

Q3	1.337(1.072–1.668)	0.01	1.440(1.149–1.807)	0.0016	1.156(0.913–1.465)	0.2277	1.133(0.892–1.441)	0.3056

Q4	1.662(1.340–2.065)	<0.001	1.901(1.518–2.384)	<0.001	1.269(0.993–1.624)	0.0566	1.240(0.963–1.598)	0.0959

P for trend		<0.001		<0.001		0.0301		0.0606

TyG-WC (per 1 SD)	1.274(1.178–1.379)	<0.001	1.287(1.189–1.395)	<0.001	1.113(1.023–1.213)	0.0135	1.108(1.014–1.212)	0.0246

TyG-WC quartile								

Q1	1(Reference)		1(Reference)		1(Reference)		1(Reference)	

Q2	1.113(0.887–1.398)	0.3534	1.114(0.885–1.401)	0.3586	1.057(0.836–1.335)	0.6418	1.043(0.824–1.319)	0.7281

Q3	1.233(0.985–1.544)	0.0676	1.251(0.997–1.571)	0.0534	1.039(0.821–1.316)	0.7481	1.005(0.792–1.277)	0.9649

Q4	1.829(1.475–2.273)	<0.001	1.851(1.488–2.306)	<0.001	1.279(1.009–1.623)	0.042	1.247(0.976–1.596)	0.078

P for trend		<0.001		<0.001		0.0557		0.1131

TyG-WHtR (per 1 SD)	1.298(1.199–1.404)	<0.001	1.257(1.159–1.364)	<0.001	1.093(1.002–1.193)	0.0443	1.087(0.993–1.191)	0.0723

TyG-WHtR quartile								

Q1	1(Reference)		1(Reference)		1(Reference)		1(Reference)	

Q2	1.246(0.990–1.570)	0.0611	1.228(0.973–1.551)	0.0846	1.117(0.881–1.419)	0.3601	1.102(0.867–1.402)	0.4252

Q3	1.404(1.119–1.763)	0.0034	1.384(1.098–1.746)	0.0059	1.131(0.889–1.439)	0.3148	1.102(0.865–1.406)	0.4323

Q4	2.049(1.647–2.555)	<0.001	1.904(1.515–2.396)	<0.001	1.313(1.026–1.682)	0.0306	1.285(0.996–1.663)	0.0544

P for trend		<0.001		<0.001		0.0372		0.0696

MetS-IR (per 1 SD)	1.246(1.156–1.343)	<0.001	1.304(1.207–1.409))	<0.001	1.124(1.033–1.223)	0.0068	1.112(1.020–1.213)	0.0158

MetS-IR quartile								

Q1	1(Reference)		1(Reference)		1(Reference)		1(Reference)	

Q2	1.034(0.824–1.298)	0.772	1.089(0.865–1.371)	0.4675	1.027(0.813–1.298)	0.8211	1.007(0.795–1.275)	0.955

Q3	1.250(1.001–1.561)	0.0486	1.367(1.091–1.717)	0.0067	1.130(0.893–1.431)	0.3087	1.097(0.864–1.394)	0.4459

Q4	1.665(1.343–2.068)	<0.001	1.879(1.505–2.349)	<0.001	1.278(1.005–1.628)	0.0456	1.233(0.965–1.577)	0.0936

P for trend		<0.001		<0.001		0.0334		0.0728

AIP (per 1 SD)	1.126(1.043–1.215)	0.0023	1.144(1.059–1.238)	0.0007	1.046(0.964–1.136)	0.2774	1.038(0.949–1.136)	0.4106

AIP quartile								

Q1	1(Reference)		1(Reference)		1(Reference)		1(Reference)	

Q2	1.131(0.904–1.414)	0.2798	1.139(0.909–1.427)	0.2579	1.097(0.872–1.382)	0.4296	1.082(0.856–1.367)	0.5095

Q3	1.416(1.140–1.762)	0.0017	1.433(1.150–1.788)	0.0014	1.235(0.984–1.553)	0.0684	1.200(0.952–1.514)	0.1235

Q4	1.270(1.019–1.583)	0.0336	1.327(1.062–1.661)	0.013	1.054(0.834–1.331)	0.6595	1.001(0.783–1.279)	0.9937

P for trend		0.0081		0.0028		0.4782		0.7089

CTI (per 1 SD)	1.144(1.060–1.234)	0.0005	1.133(1.049–1.223)	0.0015	1.016(0.936–1.103)	0.7001	1.004(0.917–1.099)	0.9281

CTI quartile								

Q1	1(Reference)		1(Reference)		1(Reference)		1(Reference)	

Q2	1.221(0.977–1.528)	0.0785	1.155(0.922–1.447)	0.2109	1.079(0.857–1.359)	0.5175	1.057(0.838–1.333)	0.6409

Q3	1.290(1.034–1.611)	0.0243	1.243(0.994–1.556)	0.0567	1.031(0.817–1.300)	0.7979	0.999(0.789–1.265)	0.9978

Q4	1.474(1.184–1.835)	0.0005	1.416(1.135–1.767)	0.002	1.079(0.855–1.361)	0.5231	1.037(0.808–1.332)	0.7741

P for trend		0.0006		0.0017		0.6352		0.9036


Model I was unadjusted; Model II included adjustments for gender and age; and Model III contained additional adjustments for marital status, smoking status, drinking status, education level, residence, hypertension, dyslipidemia, kidney disease, liver disease, antidiabetic drugs, lipid-lowering agents, antihypertensive drugs. Model IV further adjusted for TC, LDL, BUN, Creatinine, SBP, and DBP.

When examining the associations of eGDR and CVAI with specific cardiovascular outcomes, we observed similar patterns for both stroke and heart disease (Table S3). For stroke outcomes, each standard deviation increase in eGDR was associated with a 46.6% lower risk (OR = 0.534, 95% CI: 0.030–0.956, *P* = 0.0432) in the fully adjusted model (Model IV). This protective association demonstrated a strong dose-response relationship, with participants in the highest eGDR quartile showing a 92.1% lower stroke risk compared to those in the lowest quartile (OR = 0.079, 95% CI: 0.017–0.328, P = 0.0068). Conversely, each standard deviation increase in CVAI was associated with a 33.7% higher risk of stroke (OR = 1.337, 95% CI: 1.017–1.755, *P* = 0.0365). For heart disease, similar but somewhat attenuated associations were observed. Each standard deviation increase in eGDR was associated with a 20.5% lower risk of heart disease (OR = 0.795, 95% CI: 0.616–1.021, P = 0.0748), while each standard deviation increase in CVAI was associated with an 8.8% higher risk (OR = 1.088, 95% CI: 0.948–1.248, P = 0.2262), though neither reached statistical significance in the fully adjusted model.

### 3.4 Subgroup and interaction analyses

**Figure 2:**
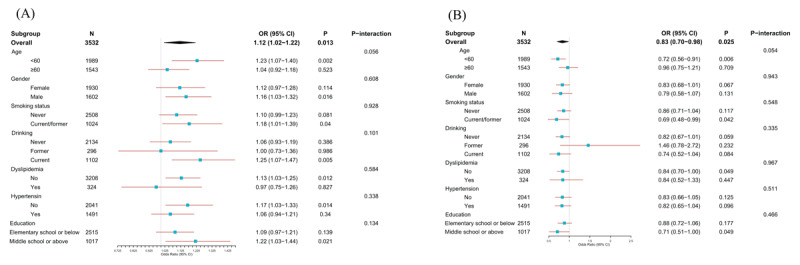
Subgroup analyses of the associations between insulin resistance indices and incident cardiovascular disease. **(A)** Forest plot showing the association between Chinese Visceral Adiposity Index (CVAI) and CVD risk across different demographic and clinical subgroups. **(B)** Forest plot illustrating the association between estimated glucose disposal rate (eGDR) and CVD risk across subgroups. No significant interactions were observed between either index and demographic or clinical characteristics (all P for interaction > 0.05), suggesting consistent associations across different subgroups.

To assess the robustness of our findings and identify potential effect modifiers, we conducted comprehensive subgroup analyses for both CVAI and eGDR in relation to incident CVD. For CVAI (Figure 2A), the positive association with CVD risk remained significant in several key subgroups, including participants aged <60 years (OR = 1.23, 95% CI: 1.07–1.40), males (OR = 1.16, 95% CI: 1.03–1.32), current/former smokers (OR = 1.18, 95% CI: 1.01–1.39), current drinkers (OR = 1.25, 95% CI: 1.07–1.47), those without dyslipidemia (OR = 1.13, 95% CI: 1.03–1.25), those without hypertension (OR = 1.17, 95% CI: 1.03–1.33), and those with middle school education or above (OR = 1.22, 95% CI: 1.03–1.44). Similarly, for eGDR (Figure 2B), the inverse association with CVD was particularly pronounced in participants aged <60 years (OR = 0.72, 95% CI: 0.56–0.91), current/former smokers (OR = 0.69, 95% CI: 0.48–0.99), those without dyslipidemia (OR = 0.84, 95% CI: 0.70–1.00), and those with middle school education or above (OR = 0.71, 95% CI: 0.51–1.00). Although we observed some variation in effect sizes across subgroups, no significant interactions were detected between either index and age, gender, smoking status, drinking status, dyslipidemia, hypertension, or education level (all *P* for interaction > 0.05), suggesting that the associations of CVAI and eGDR with CVD risk were relatively consistent across different demographic and clinical characteristics. To further validate the robustness of our findings, we conducted multiple sensitivity analyses. The significant associations between eGDR, CVAI, and incident CVD remained consistent after excluding participants with incomplete covariate data (Table S4), those who developed CVD in wave 2 during follow-up (Table S5), and those receiving antihyperglycemic, antihypertensive, or lipid-lowering treatments at baseline (Table S6). Furthermore, E-value analysis supported the robustness of our findings against potential unmeasured confounding. The E-values for the association between eGDR and incident CVD were 1.44 for the point estimate and 1.14 for the confidence interval limit (Figure S3A), while for CVAI, the E-values were 1.31 and 1.13, respectively (Figure S3B). These results indicate that only an unmeasured confounder with risk ratios exceeding these E-values for both exposure and outcome associations could nullify the observed relationships, suggesting that our findings are relatively robust to unmeasured confounding of moderate strength.

To evaluate the consistency of our findings across different glycemic states, we performed stratified analyses separating participants into prediabetes (n = 2622) and diabetes (n = 910) subgroups. In the prediabetes population (Figure S4A), the KNN algorithm maintained excellent discriminative performance with an AUC of 0.937 (95% CI: 0.929–0.946), demonstrating its superior capability in identifying individuals at high cardiovascular risk even at the earliest stage of glucose dysregulation. Similarly, in the diabetes population (Figure S4B), the KNN algorithm demonstrated robust and comparable predictive capability with an AUC of 0.934 (95% CI: 0.925–0.944), indicating that the model’s discriminative ability remains excellent even in patients with established diabetes. These parallel findings across both glycemic states highlight the robustness and generalizability of our ML approach for cardiovascular risk stratification across the entire spectrum of abnormal glucose metabolism.

To ensure proper model validation, the entire dataset (n = 3,532) was randomly split into training and validation sets using a 1:1 ratio as a sensitivity analysis. The training set (n = 1,766) was used for model development, hyperparameter tuning, and feature selection, while the validation set (n = 1,766) was held out exclusively for independent performance evaluation. Train-test split validation demonstrated excellent and consistent model performance across both datasets. In the training set (Figure S5A), the KNN algorithm achieved an AUC of 0.938 (95% CI: 0.927–0.948), while maintaining robust performance in the independent validation set with an AUC of 0.932 (95% CI: 0.921–0.942) (Figure S5B). Similarly, LightGBM demonstrated strong performance with a training AUC of 0.918 (95% CI: 0.903–0.933) and a validation AUC of 0.902 (95% CI: 0.884–0.920). The minimal differences in AUC values between training and validation sets confirm the absence of overfitting and demonstrate excellent model stability.

### 3.5 Dose-response relationship between eGDR and CVAI and new-onset CVD

**Figure 3:**
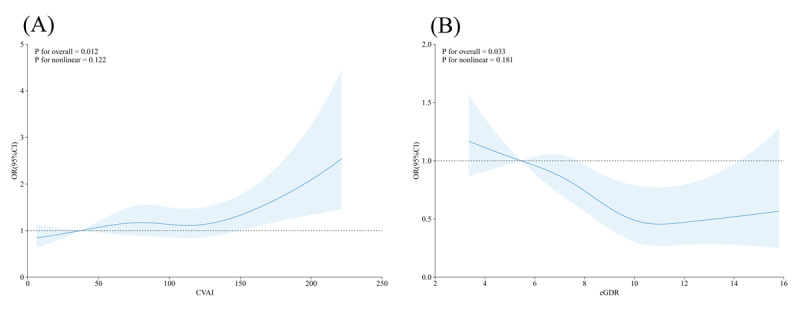
Dose-response relationships between insulin resistance indices and risk of incident cardiovascular disease. **(A)** Restricted cubic spline analysis demonstrating the relationship between Chinese Visceral Adiposity Index (CVAI) and the odds ratio of new-onset cardiovascular disease. **(B)** Restricted cubic spline analysis illustrating the relationship between estimated glucose disposal rate (eGDR) and the odds ratio of new-onset cardiovascular disease.

To further elucidate the nature of the relationships between eGDR and CVAI and CVD risk, we performed RCS with comprehensive covariate adjustment. After controlling for variables in the fully adjusted model, we observed significant associations for both indices. For CVAI (Figure 3A), a significant positive dose-response relationship with CVD risk was evident (*P* for overall = 0.012), with no significant evidence of nonlinearity (*P* for nonlinear = 0.122). This suggests that the risk of CVD increases in a linear fashion with higher CVAI values, with no threshold effect. Similarly, for eGDR (Figure 3B), a significant inverse dose-response relationship with CVD risk was observed (P for overall = 0.033), also without significant nonlinearity (*P* for nonlinear = 0.181). This indicates that higher eGDR values (reflecting better insulin sensitivity) are associated with progressively lower CVD risk in a linear pattern. These findings further support the robustness of CVAI and eGDR as potential biomarkers for CVD risk stratification in patients with prediabetes or diabetes.

### 3.6 Feature selection in machine learning

**Figure 4:**
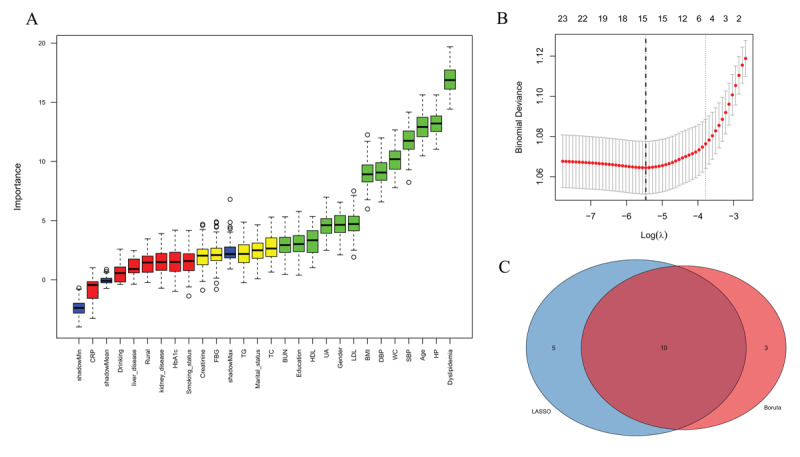
Feature selection for cardiovascular disease risk prediction using machine learning approaches. **(A)** The Boruta algorithm identified 13 features as important predictors of CVD risk. **(B)** Feature selection using LASSO (Least Absolute Shrinkage and Selection Operator) regression with ten-fold cross-validation. The plot shows the binomial deviance against log(λ), where λ is the regularization parameter. The numbers above the plot indicate the number of non-zero coefficients retained in the model at each value of λ. The optimal model using the minimum lambda criterion identified 15 key variables. **(C)** Venn diagram showing the intersection of features selected by both Boruta and LASSO methods, resulting in 10 final variables (gender, education, dyslipidemia, hypertension, UA, WC, BUN, DBP, age, and BMI) that were subsequently used in the machine learning models for predicting CVD incidence.

To identify the most significant variables for predicting CVD risk, we employed two complementary feature selection methods: the Boruta algorithm and LASSO regression (Table S7). The Boruta algorithm, known for its robustness in identifying all relevant features, selected 13 variables as important predictors of CVD (Figure 4A). LASSO regression with ten-fold cross-validation was performed to identify the most parsimonious set of predictors while maintaining model performance. Through iterative analysis using the minimum lambda criterion, LASSO identified 15 key variables (Figure 4B). To enhance the robustness of our feature selection and minimize the risk of both false positives and false negatives, we used the intersection of these two methods, resulting in a final set of 10 variables: gender, education, dyslipidemia, hypertension, UA, WC, BUN, DBP, age, and BMI (Figure 4C). These variables were subsequently utilized in all our machine learning models for predicting CVD incidence.

### 3.7 Comparison of nine machine learning models

**Figure 5:**
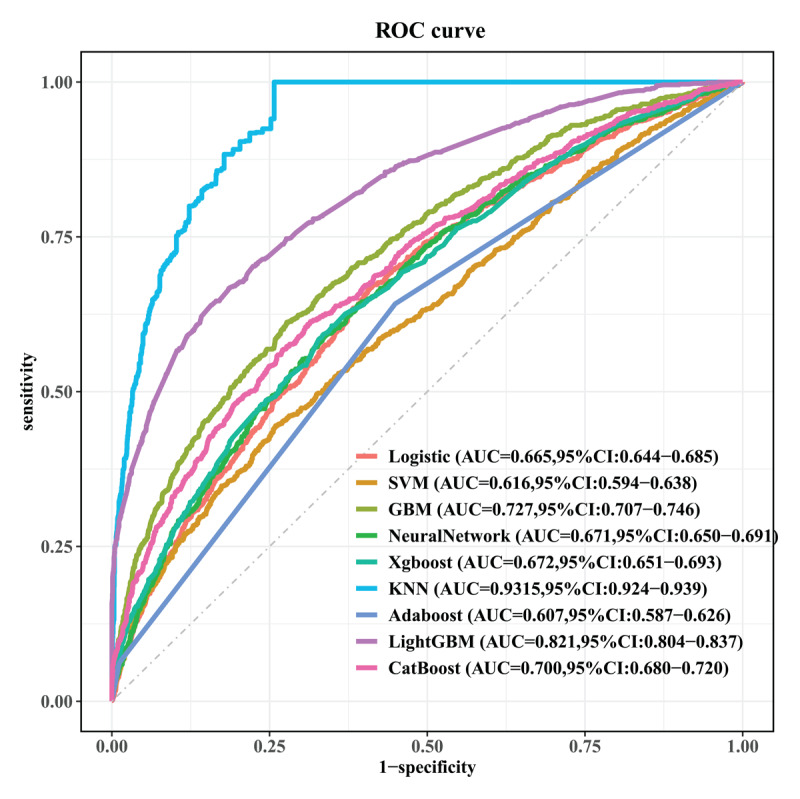
Performance comparison of nine machine learning algorithms for predicting cardiovascular disease incidence. The receiver operating characteristic (ROC) curves illustrate the discriminative ability of each model in identifying individuals at risk of developing cardiovascular disease. The K-Nearest Neighbors (KNN) algorithm demonstrated markedly superior performance with the highest area under the curve (AUC) of 0.9315 (95% CI: 0.924–0.939), indicating excellent discrimination. All models were trained using the final set of 10 selected features identified through the combined Boruta and LASSO feature selection process.

To evaluate the predictive performance of different algorithms for CVD risk prediction, we developed and compared nine distinct machine learning models (Figure 5). Among all models tested, the KNN algorithm demonstrated markedly superior performance with the highest AUC of 0.9315 (95% CI: 0.924–0.939), indicating excellent discriminative ability for identifying individuals at risk of developing CVD. The LightGBM algorithm showed the second-best performance with an AUC of 0.821 (95% CI: 0.804–0.837), followed by GBM with an AUC of 0.727 (95% CI: 0.707–0.746), CatBoost (AUC = 0.700, 95% CI: 0.680–0.720), Neural Network (AUC = 0.671, 95% CI: 0.650–0.691), Xgboost (AUC = 0.672, 95% CI: 0.651–0.693), Logistic Regression (AUC = 0.665, 95% CI: 0.644–0.685), SVM (AUC = 0.616, 95% CI: 0.594–0.638), and AdaBoost (AUC = 0.607, 95% CI: 0.587–0.626).

### 3.8 Incremental predictive value of the eGDR and CVAI index for CVD

**Figure 6:**
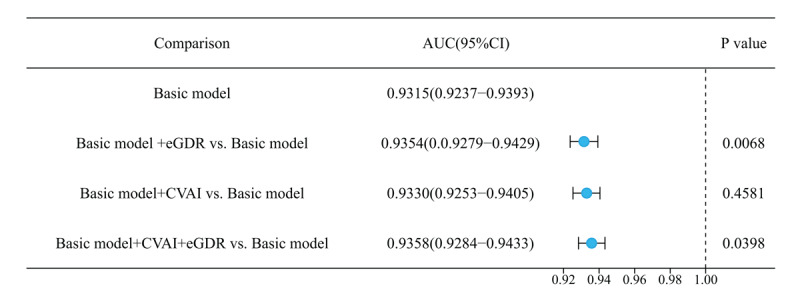
Incremental predictive value of insulin resistance indices when added to the baseline model for cardiovascular disease prediction. This forest plot demonstrates the improvement in discriminative ability when eGDR and CVAI were incorporated into the K-Nearest Neighbors (KNN) algorithm, which showed superior performance among all machine learning approaches.

After establishing the performance of our basic predictive models incorporating traditional risk factors (gender, education, dyslipidemia, hypertension, UA, WC, BUN, DBP, age, and BMI), we further evaluated whether the addition of eGDR and CVAI could enhance the predictive capabilities of these models. To assess this incremental value, we developed three additional model variations for each of the nine machine learning algorithms: one incorporating eGDR alone, another with CVAI alone, and a third model integrating both indices simultaneously with the baseline risk factors. The results demonstrated a modest but statistically significant improvement in predictive performance when IR indices were added to the basic model (Figure 6). The basic KNN model achieved an AUC of 0.9315 (95% CI: 0.9237–0.9393). When eGDR was incorporated, the AUC significantly increased to 0.9354 (95% CI: 0.9279–0.9429, *P* = 0.0068), highlighting its independent predictive value beyond traditional risk factors. The addition of CVAI resulted in a slight improvement (AUC = 0.9330, 95% CI: 0.9253–0.9405, *P* = 0.4581), while the model incorporating both indices demonstrated the highest performance (AUC = 0.9358, 95% CI: 0.9284–0.9433, *P* = 0.0398) (Figure S6).

### 3.9 Comparison of multiple modified machine learning models

**Figure 7:**
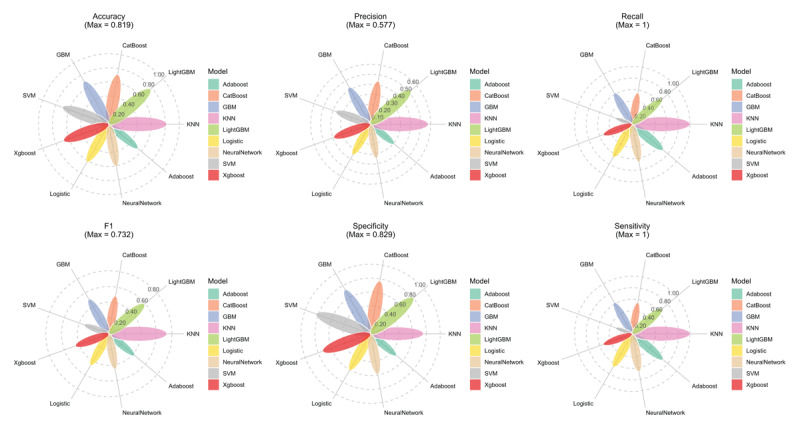
Comprehensive evaluation of performance metrics across nine machine learning models incorporating eGDR and CVAI for cardiovascular disease prediction. This multi-panel figure displays six key performance metrics for each model: **(A)** Accuracy; **(B)** Precision; **(C)** Recall; (D) F1 score; **(E)** Specificity; and **(F)** Sensitivity.

We further compared the modified machine learning model performance, which incorporated the eGDR and CVAI index. Feature importance analysis from the optimized KNN model revealed that eGDR and CVAI were the two most important predictors for CVD incidence, with importance scores substantially higher than traditional risk factors (Figure S7). Figure 7 illustrates the comprehensive performance metrics of all nine machine learning models after enhancement with eGDR and CVAI. The models demonstrated varying performance across different evaluation metrics (Table S8). The KNN model consistently outperformed all other algorithms across multiple performance metrics. It achieved the highest accuracy (0.82), precision (0.58), recall (1.00), and F1 score (0.73), indicating superior balanced performance in both correctly identifying positive cases and avoiding false positives. Additionally, the KNN model demonstrated excellent sensitivity (1.00) and specificity (0.76), highlighting its effectiveness in both detecting true positive CVD cases and correctly identifying non-CVD cases. Figure S8A shows DCA, demonstrating that the KNN model consistently provides the highest net clinical benefit across a wide range of threshold probabilities compared to all other models. The calibration curves of the nine models are shown in Figure S8B, with the KNN model exhibiting excellent calibration with minimal deviation between predicted probabilities and observed event rates, particularly in the clinically relevant mid-range probability zones.

#### Model Interpretability Analysis

SHAP analysis was performed to elucidate the contribution of individual features to the KNN model’s predictions. The beeswarm plot (Figure S9) revealed that eGDR and CVAI were the two most influential predictors of cardiovascular disease risk, demonstrating the largest absolute SHAP values and greatest impact on model output. Lower eGDR values (indicating greater insulin resistance) consistently contributed to higher cardiovascular disease risk predictions, as evidenced by the clustering of low eGDR values (blue points) with positive SHAP values. Conversely, higher CVAI values (reflecting greater visceral adiposity dysfunction) were associated with positive SHAP values, confirming their role in increasing cardiovascular risk. The waterfall plot (Figure S10) illustrates an individual-level prediction example, showing how specific feature values combine to generate personalized cardiovascular risk predictions. For instance, in a representative patient, low eGDR (12.1 mg/kg/min) contributed +0.0881 to the SHAP value (increasing risk), while low CVAI (54.9) contributed –0.0132 (reducing risk). The cumulative effect of all features resulted in the final individualized risk prediction, demonstrating the model’s capability for personalized risk stratification.

## 4. Discussion

This comprehensive longitudinal study examined ten IR surrogate indices for predicting new-onset CVD in Chinese adults with prediabetes or diabetes. Among the indices evaluated, eGDR and CVAI demonstrated superior performance. Each standard deviation increase in eGDR was associated with 17.8% lower CVD risk, while each standard deviation increase in CVAI corresponded to 12.4% higher risk. These associations persisted across subgroup and sensitivity analyses. Restricted cubic spline analyses revealed significant linear dose-response relationships without threshold effects for both indices. Incorporating eGDR and CVAI into machine learning algorithms significantly enhanced predictive capability beyond traditional risk factors, with the KNN algorithm achieving excellent discrimination (AUC: 0.936, 95% CI: 0.928–0.943). These findings suggest eGDR and CVAI could serve as valuable, accessible tools for cardiovascular risk stratification in individuals with abnormal glucose metabolism.

The eGDR index, initially validated in type 1 diabetes patients (27), incorporates three clinically accessible parameters: HbA1c, waist-to-hip ratio, and hypertension status. Our findings extend previous observations of eGDR’s predictive capability for CVD events to Chinese individuals with prediabetes or diabetes. Zhang et al. (28) reported eGDR as the strongest predictor of stroke and heart disease in the CHARLS cohort (AUC: 0.716). Our analysis advances this understanding by demonstrating eGDR’s superiority over nine other IR indices in machine learning models, achieving substantially higher discrimination (AUC: 0.936). Additionally, we identified CVAI as an independent CVD predictor, a marker not evaluated in previous CHARLS-based studies. Our machine learning approach addressed limitations of conventional regression models. The KNN algorithm captured nonlinear interactions between IR indices and traditional risk factors, achieving significantly higher discrimination than the AUC of 0.712 reported in recent nationwide CHARLS analyses (7). We identified linear dose-response relationships for both eGDR and CVAI, indicating that modest improvements in insulin sensitivity may yield cardiovascular benefits, contrasting with threshold effects reported by Zabala et al. (29) in type 2 diabetes populations. The integration of feature selection techniques (Boruta and LASSO) identified the most impactful predictors while minimizing overfitting, representing a methodological advancement over traditional approaches such as the Cox regression models used by Lu et al. (30) for predicting acute ischemic stroke outcomes. Mechanistically, unlike indices primarily reflecting hepatic insulin resistance, eGDR captures insulin action across multiple tissues, potentially explaining its superior performance for systemic vascular complications (31,32). The eGDR components—glycemic control, central adiposity, and blood pressure—represent critical pathways through which insulin resistance contributes to atherogenesis, including endothelial dysfunction, vascular inflammation, and abnormal lipid metabolism (33).

CVAI, specifically developed for Chinese populations, incorporates age, BMI, waist circumference, and metabolic parameters to estimate visceral adiposity and insulin resistance (34). Our findings of CVAI’s strong positive association with CVD risk align with emerging evidence. Yang et al. (35) demonstrated that high CVAI was independently associated with elevated CVD, coronary heart disease, and stroke risks (HR = 1.61, 95% CI: 1.42–1.83). Liu et al. (36) found CVAI demonstrated superior predictive performance for cardiovascular risk in postmenopausal women during a 10-year follow-up, while a Chinese Multi-Ethnic Cohort study confirmed excellent predictive performance in type 2 diabetes patients (37). Zhang et al. (38) demonstrated CVAI’s association with increased stroke incidence in middle-aged and elderly Chinese populations, complementing our findings. Mechanistically, visceral adiposity contributes to cardiovascular risk through multiple pathways: enhanced lipolytic activity releasing free fatty acids that promote hepatic insulin resistance and dyslipidemia (39); secretion of pro-inflammatory adipokines inducing systemic inflammation and endothelial dysfunction (40); and activation of the renin-angiotensin-aldosterone system contributing to hypertension and vascular remodeling (40). CVAI’s advantage over traditional anthropometric measures stems from incorporating age- and sex-specific factors, accounting for differential impacts of adiposity patterns across demographic groups (34), and its population-specific derivation may better capture unique body composition characteristics in Chinese populations compared to Western-derived indices (41).

Among nine machine learning algorithms evaluated, KNN demonstrated markedly superior performance (AUC: 0.936) compared to logistic regression (AUC: 0.665) and SVM (AUC: 0.616). This exceptional performance suggests cardiovascular risk prediction in individuals with prediabetes/diabetes may be better suited to instance-based learning approaches that capture complex, non-linear interactions without requiring explicit specification of functional relationships (42,43). Feature importance analysis identified eGDR and CVAI as the two most important predictors, with scores substantially higher than traditional risk factors, validating their biological and clinical significance in cardiovascular pathophysiology. The incremental predictive value when adding these indices to traditional risk factors highlights their potential to enhance existing risk prediction frameworks. Decision curve analysis demonstrated that the KNN model incorporating eGDR and CVAI consistently provided the highest net clinical benefit across threshold probabilities, suggesting meaningful utility for informing preventive interventions. These findings highlight the potential of combining novel biomarkers with advanced analytical approaches to improve risk stratification in high-risk populations.

The robust associations between IR indices and cardiovascular outcomes reflect IR’s central role in atherosclerosis pathophysiology through several interconnected mechanisms. IR promotes endothelial dysfunction through reduced nitric oxide production, increased endothelin-1 secretion, and enhanced adhesion molecule expression (44); drives dyslipidemia characterized by elevated triglycerides, reduced HDL cholesterol, and increased small dense LDL particles that undergo oxidative modification (45); and enhances sympathetic nervous system activity and sodium retention, promoting hypertension and adverse vascular remodeling (46). These mechanisms may operate differently across glucose dysregulation stages. In prediabetes, compensatory hyperinsulinemia primarily drives lipid abnormalities and endothelial dysfunction (47,48), while in established diabetes, hyperglycemia-induced oxidative stress and advanced glycation end-products exacerbate vascular damage (49,50). The stronger associations observed for stroke than heart disease may reflect the particular importance of hypertension and small vessel disease in cerebrovascular pathology (51). The linear dose-response relationships for both eGDR and CVAI suggest cardiovascular risk increases progressively with worsening IR without threshold effects, highlighting the importance of addressing IR as a continuous risk factor.

Our findings offer practical applications for cardiovascular risk management in patients with prediabetes or diabetes. First, eGDR and CVAI can be easily calculated using routinely collected parameters (waist circumference, BMI, blood pressure, HbA1c, and lipid profiles) without additional specialized testing or costs, making them suitable for primary care and resource-limited settings. Second, our KNN algorithm (AUC = 0.932) could be integrated into electronic health record systems as a clinical decision support tool to identify high-risk patients requiring intensive lifestyle modifications, pharmacological interventions, or frequent cardiovascular surveillance. Third, these metabolic indices may facilitate personalized risk stratification beyond traditional cardiovascular risk scores in populations with dysglycemia where IR represents a central pathophysiological mechanism. Future research should focus on implementation science to facilitate translation into clinical practice.

Several limitations should be acknowledged. First, definitions of diabetes, CVD, hypertension, and dyslipidemia relied on self-reported physician diagnosis and medication use, which may introduce misclassification bias. Medications are increasingly prescribed beyond traditional indications: GLP-1 receptor agonists for weight management in non-diabetic individuals, statins for primary prevention without diagnosed dyslipidemia, and antihypertensives for cardiovascular or renal protection in normotensive patients. Self-reported diagnoses are also subject to recall bias and may underestimate disease prevalence, particularly in populations with limited healthcare access. Second, we could not differentiate CVD subtypes, particularly ischemic versus hemorrhagic stroke and specific cardiac conditions. Given insulin resistance’s stronger association with atherosclerotic disease, our findings likely primarily reflect ischemic cardiovascular events, which constitute the majority of CVD cases in Chinese populations (52). Third, we lacked external validation in an independent cohort. While rigorous internal validation using train-test split demonstrated robust performance on held-out data, external validation is essential for confirming generalizability. Our performance estimates should be interpreted as applicable to populations similar to CHARLS participants (Chinese adults aged ≥45 years with prediabetes or diabetes). Fourth, substantial participant exclusion due to missing data may introduce selection bias if missingness is not completely at random. Future studies employing multiple imputation could enhance generalizability and reduce selection bias.

## 5. Conclusion

Our comprehensive evaluation of ten IR surrogate indices demonstrated that eGDR and CVAI exhibit superior predictive performance for new-onset CVD in Chinese adults with prediabetes or diabetes. eGDR showed a robust inverse association with cardiovascular risk, while CVAI demonstrated a positive relationship, both following linear dose-response patterns without threshold effects. The incorporation of these indices into machine learning algorithms, particularly the KNN model, significantly enhanced predictive capability beyond traditional risk factors, achieving excellent discrimination for identifying individuals at high risk of cardiovascular events. These findings have important clinical implications, suggesting that eGDR and CVAI could serve as valuable, easily accessible tools for early identification of high-risk individuals in routine clinical practice. Further research is warranted to validate these findings in diverse populations and to develop simplified clinical prediction tools that can translate these insights into improved patient care and outcomes.

## Data Accessibility Statement

The datasets used in this investigation are available in online repositories. Detailed descriptions of each survey and corresponding data have been published at http://charls.pku.edu.cn//.

## Additional File

The additional file for this article can be found as follows:

10.5334/gh.1532.s1 Supplementary Materials. Figures S1 to S10 and Tables S1 to S8.
