# Supplementary material for: Assessment of Ten Insulin Resistance Surrogate Indexes Predicts New-Onset Cardiovascular Disease Incidence in Patients with Prediabetes or Diabetes: Insights from CHARLS Data with Machine Learning Analysis
Source: Glob Heart. 2026 Mar 12;21(1):17. doi: 10.5334/gh.1532 (PMC12985947; doi:10.5334/gh.1532)
Supplement: Supplementary Materials. — Figures S1 to S10 and Tables S1 to S8. [file gh-21-1-1532-s1.pdf]

Assessment of ten insulin resistance surrogate indexes predicts new-onset cardiovascular disease incidence in patients with prediabetes or diabetes: insights from CHARLS data with machine learning analysis

Hang Xie[1*], Chaoying Yan[2], Yi Zheng[3], Haoyu Wu[1]

[1]Department of Cardiovascular Medicine, The First Affiliated Hospital of Xi'an Jiaotong University, Xi'an 710061, Shaanxi, China

[2]Department of Anesthesiology, The First Affiliated Hospital of Xi'an Jiaotong University, 710061, Xi'an, Shaanxi, China;

[3]Department of Dermatology, The Second Affiliated Hospital of Xi'an Jiaotong University, Xi'an, China

Supplementary Materials

I. Definition of ten insulin resistance (IR) surrogate indices

The methods for calculating ten IR indices are as follows:

(

1

(2) TyG-BMI = TyG * BMI (PMID: 34488806);

)

(3) TyG-WC = TyG * WC (PMID: 39955571);

(4) TyG-WHtR = TyG * WHtR (PMID: 38992634);

r

(

i

5

(6) Atherogenic index of plasma (AIP) = log($TG_{mg/dl}$ /$HDL\text{-}C_{mg/dl}$) (PMID: 39915878);

g

)

(7) Triglyceride high-density cholesterol-glucose body index (TyHGB) = TG/HDL-C + 0.7*$FBG_{mmol/L}$ + 0.1*BMI kg/m2 (PMID: 39956901);

M

c

e

8

(9) estimated glucose disposal rate (eGDR) = 21.158 − 0.09 * WC − 3.407 *hypertension $_{(yes = 1/no = 0)}$ − 0.551 * HbA1c% (PMID: 40241176);

i

b

(10) Chinese Visceral Adiposity Index (CVAI) for male = − 267.93 + 0.68 × Age_{years} +0.03 * BMI_{kg/m^2} + 4.00 * WC_{cm} + 22.00 * log10($TG_{mmol/L}$) − 16.32 * $HDL_{mmol/L}$; For female, CVAI = − 187.32 + 1.71 * Age_{years}+ 4.23 * BMI_{kg/m^2} + 1.12 * WC_{cm} + 39.76 * log10($TG_{mmol/L}$) − 11.66 * $HDL_{mmol/L}$ (PMID: 40075466);

g

l

a

u

c

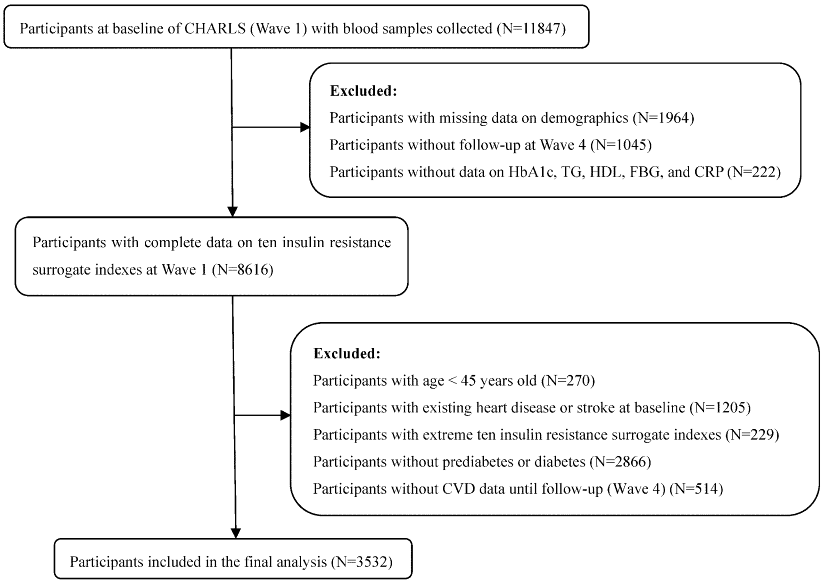

Figure S1. Flowchart of participant selection in the CHARLS cohort study. CHARLS, China Health and Retirement Longitudinal Study; CVD, cardiovascular disease; TG, triglyceride; HDL, high-density lipoprotein; FBG, fasting blood glucose; HbA1c, glycosylated hemoglobin A1c; CRP, C-reactive protein.

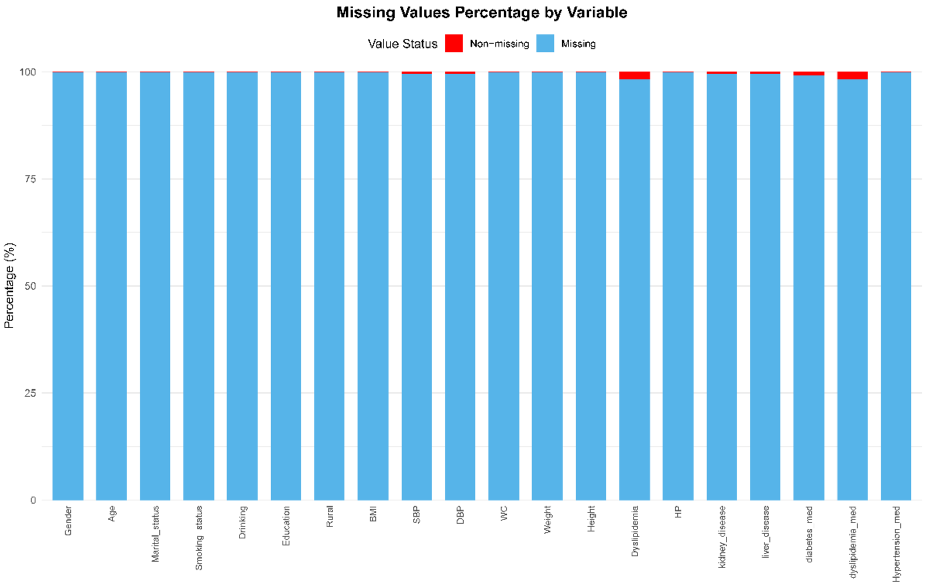

Figure S2. Distribution of missing values across study variables. Bar chart showing the percentage of missing data for each variable included in the analysis. The blue bars represent the proportion of non-missing values, while the red bars indicate missing values for each variable. Most variables demonstrated less than 5% missing data, with the highest rates of missingness observed in medication-related variables (hypertension medication, diabetes medication, and dyslipidemia medication), kidney disease, liver disease, and certain laboratory measurements. Gender, age, and marital status showed near-complete data collection. The overall missing data rate was approximately 3.8% of total data points across all variables.

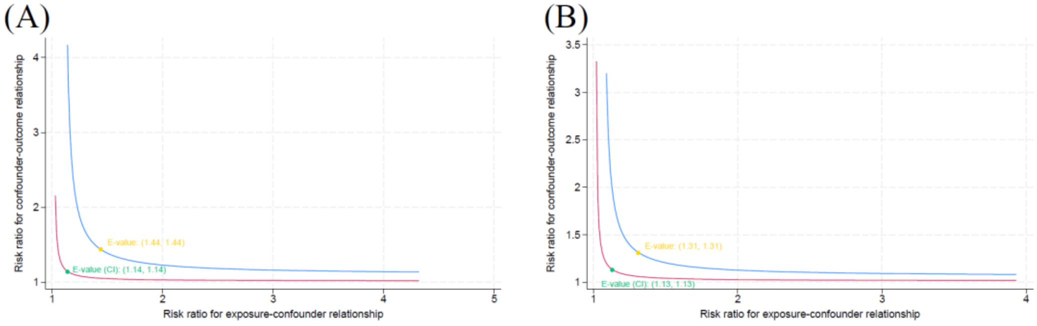

Figure S3. E-value sensitivity analysis for unmeasured confounding. (A) E-value contour plot for the association between eGDR and incident CVD. The plot illustrates the strength of unmeasured confounding (defined by the risk ratio for both exposure-confounder and confounder-outcome relationships) required to nullify the observed association. E-values were 1.31 for the point estimate and 1.13 for the confidence interval limit. (B) E-value contour plot for the association between CVAI and incident CVD. The plot demonstrates the minimum strength of association that an unmeasured confounder would need to have with both CVAI and incident CVD to fully explain away the observed relationship. E-values were 1.44 for the point estimate and 1.14 for the confidence interval limit. Higher E-values indicate greater robustness to unmeasured confounding.

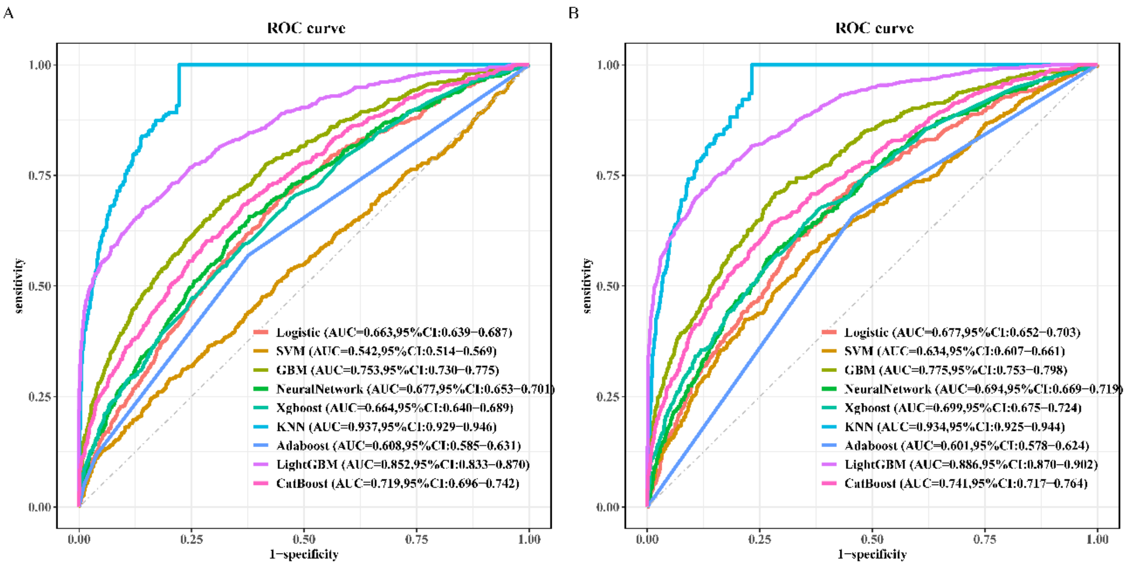

Figure S4 Subgroup Analysis by Glucose Tolerance Status. A and B present the ROC curves for all nine machine learning models in prediabetes and diabetes populations, respectively. In the prediabetes subgroup, the KNN algorithm achieved the highest discriminative performance with an AUC of 0.937 (95% CI: 0.929-0.946), followed by LightGBM (AUC=0.852, 95% CI: 0.833-0.870) and GBM (AUC=0.753, 95% CI: 0.730-0.775). Similarly, in the diabetes subgroup, KNN demonstrated superior performance with an AUC of 0.934 (95% CI: 0.925-0.944), followed by LightGBM (AUC=0.886, 95% CI: 0.870-0.902) and GBM (AUC=0.775, 95% CI: 0.753-0.798).

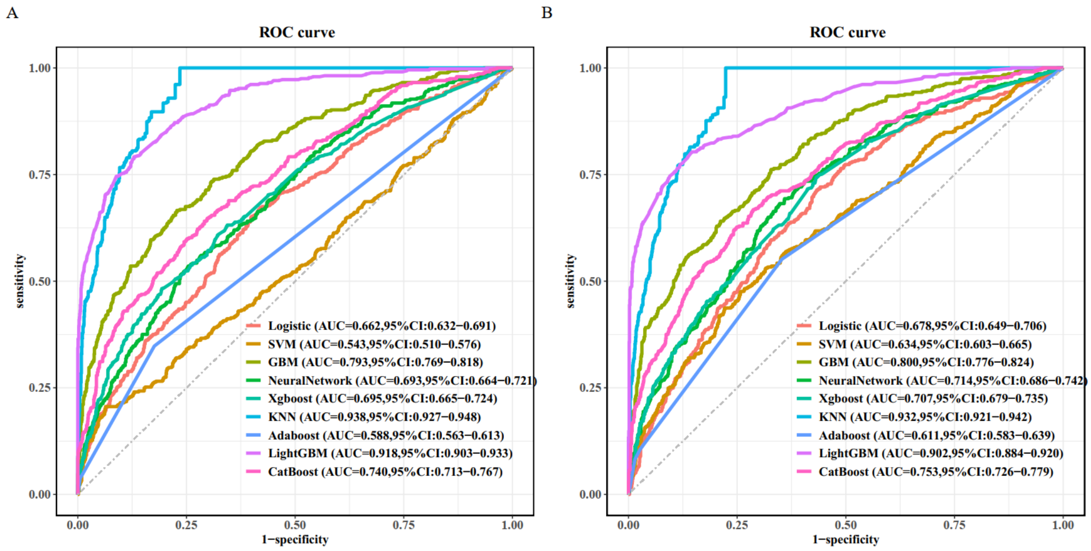

Figure S5 Receiver Operating Characteristic (ROC) Curves of Nine Machine Learning Algorithms for Predicting Cardiovascular Disease in Training and Validation Sets. ROC curves demonstrating the discriminative performance of nine machine learning algorithms in both training (n=1,766) and validation (n=1,766) sets. (A) Training set performance: KNN achieved the highest area under the curve (AUC = 0.938, 95% CI: 0.927-0.948), followed by LightGBM (AUC = 0.918, 95% CI: 0.903-0.933) and GBM (AUC = 0.793, 95% CI: 0.769-0.818). (B) Validation set performance: KNN maintained excellent discriminative capability (AUC = 0.932, 95% CI: 0.921-0.942), followed by LightGBM (AUC = 0.902, 95% CI: 0.884-0.920) and GBM (AUC = 0.800, 95% CI: 0.776-0.824). The minimal differences in AUC values between training and validation sets confirm the absence of overfitting and demonstrate robust model generalizability. The diagonal reference line represents random classification (AUC = 0.50).

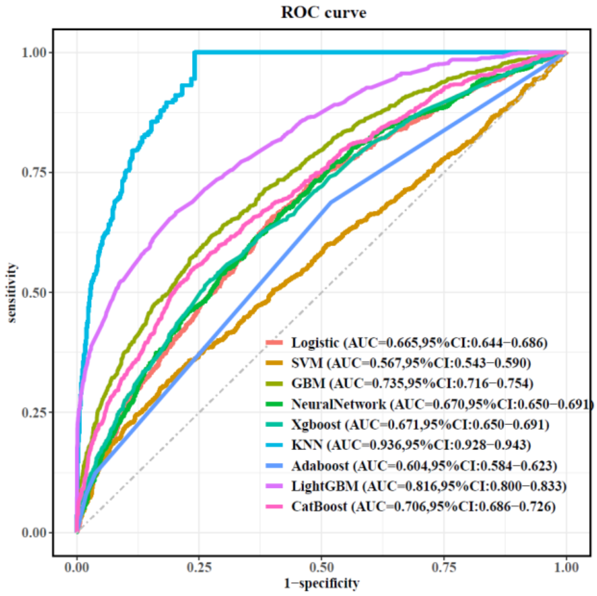

Figure S6. ROC curves of nine machine learning models incorporating both eGDR and CVAI for predicting CVD incidence. Receiver operating characteristic (ROC) curves comparing the discriminative ability of nine different machine learning algorithms after incorporating both eGDR and CVAI indices along with traditional risk factors. The K-Nearest Neighbors (KNN) algorithm

demonstrated superior performance with the highest AUC of 0.936 (95% CI: 0.928-0.943), followed by LightGBM (AUC = 0.816, 95% CI: 0.800-0.833) and Gradient Boosting Machine (GBM) (AUC = 0.735, 95% CI: 0.716-0.754). The enhanced KNN model incorporating both indices showed a significant improvement in predictive performance compared to the basic model (AUC = 0.9358, 95% CI: 0.9284-0.9433, P = 0.0398). ROC, receiver operating characteristic; AUC, area under the curve; CI, confidence interval; eGDR, estimated glucose disposal rate; CVAI, Chinese visceral adiposity index; CVD, cardiovascular disease.

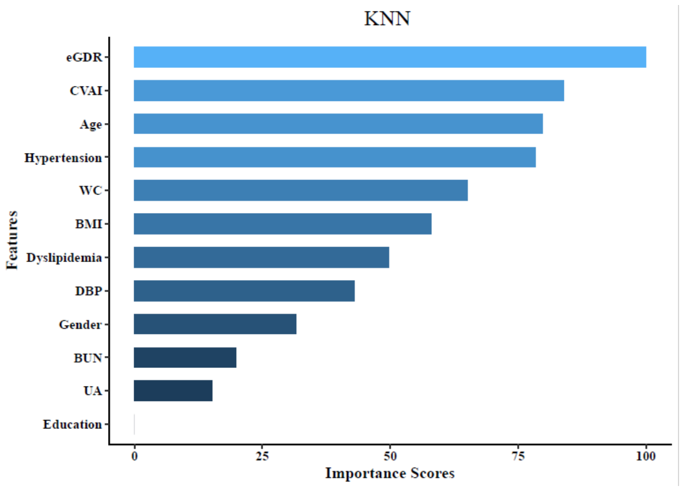

Figure S7. Feature importance ranking in the optimized KNN model for predicting CVD incidence. Relative importance of predictors in the K-Nearest Neighbors (KNN) model incorporating both eGDR and CVAI along with traditional risk factors. The eGDR and CVAI indices emerged as the two most influential predictors with substantially higher importance scores compared to conventional risk factors. This analysis highlights the significant contribution of insulin resistance surrogate indices to the model's predictive capability for cardiovascular disease. Feature importance was calculated based on permutation importance method, which measures the decrease in model performance when each feature is randomly shuffled. KNN, K-Nearest Neighbors; eGDR, estimated glucose disposal rate; CVAI, Chinese visceral adiposity index; BMI, body mass index; WC, waist circumference; DBP, diastolic blood pressure; UA, uric acid; BUN, blood urea nitrogen.

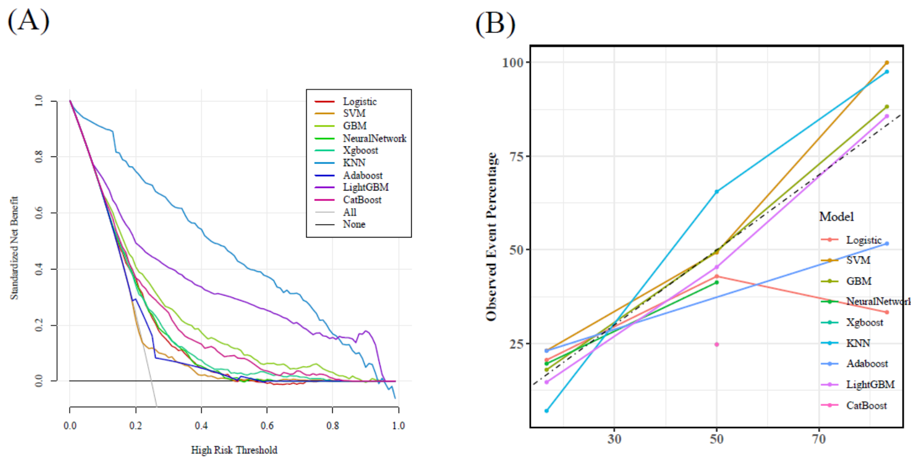

Figure S8. Clinical utility and calibration assessment of the modified machine learning models incorporating eGDR and CVAI.

(A) Decision curve analysis comparing the net benefit of nine machine learning models across different threshold probabilities. The KNN model consistently demonstrated superior net benefit across a wide range of threshold probabilities, indicating greater clinical utility for decision-making. The "All" line represents the strategy of treating all patients as high-risk, while the "None" line represents treating no patients. Models above these reference lines provide clinical benefit at the corresponding threshold probability. (B) Calibration plots showing the agreement between predicted and observed CVD events. The x-axis represents the bin midpoint of predicted probability, while the y-axis shows the actual observed event percentage. The KNN model demonstrated excellent calibration with close alignment between predicted probabilities and observed events, followed by LightGBM and GBM. A model with perfect calibration would follow the diagonal line, indicating that predicted probabilities match observed event rates. eGDR, estimated glucose disposal rate; CVAI, Chinese visceral adiposity index; KNN, K-Nearest Neighbors; GBM, Gradient Boosting Machine; SVM, Support Vector Machine; CVD, cardiovascular disease.

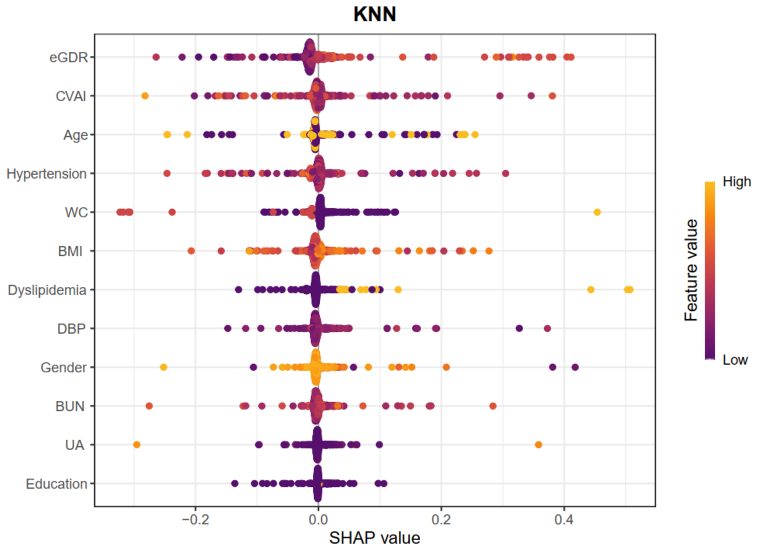

Figure S9. SHAP Beeswarm Plot Showing Feature Importance and Impact Direction for the KNN Model. Beeswarm plot displaying SHAP (SHapley Additive exPlanations) values for all features in the KNN model across the entire dataset. Each point represents an individual sample, with the x-axis showing the SHAP value (magnitude and direction of impact on prediction) and

the y-axis showing features ranked by importance. Point color indicates feature value (red = high, blue = low). eGDR and CVAI demonstrated the greatest influence on cardiovascular disease predictions. Lower eGDR values (blue points) clustered with positive SHAP values, indicating increased cardiovascular risk with greater insulin resistance. Higher CVAI values (red points) were associated with positive SHAP values, confirming increased risk with greater visceral adiposity dysfunction. This analysis provides transparent insights into feature contributions and their directional relationships with cardiovascular disease risk.

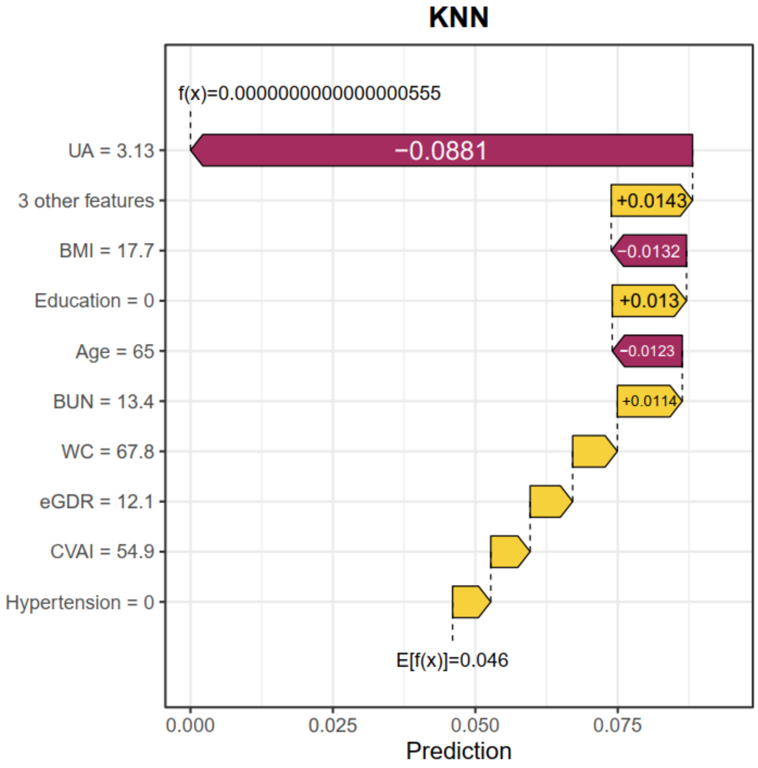

Figure S10. SHAP Waterfall Plot Illustrating Individual-Level Prediction Explanation. Waterfall plot demonstrating how individual features contribute to a specific patient's cardiovascular disease risk prediction in the KNN model. The plot shows the progression from the base value (E[f(x)] = 0.046, representing the average model output) to the final prediction (f(x) = 0.0000000000000000555) through cumulative feature contributions. Red bars indicate features increasing risk prediction, while blue bars show features decreasing risk. For this representative patient, low eGDR (12.1 mg/kg/min) substantially increased risk (+0.0881), while other features such as low CVAI (54.9) and absence of hypertension provided modest protective effects. This individualized explanation demonstrates the model's capability for transparent, interpretable risk stratification at the patient level

Table S1 The baseline characteristics stratified by quartiles of CVAI

Variables	Total (n = 3532)	Q1 (n = 883)	Q2 (n = 883)	Q3 (n = 883)	Q4 (n = 883)	P
HbA1c, Mean ± SD	5.41 ± 0.91	5.27 ± 0.78	5.35 ± 0.89	5.43 ± 0.92	5.62 ± 0.99	<0.01
FBG, Mean ± SD	120.89 ± 38.66	116.26 ± 35.79	118.68 ± 38.38	121.15 ± 36.14	127.49 ± 43.05	<0.01
UA, Mean ± SD	4.46 ± 1.25	4.27 ± 1.20	4.21 ± 1.14	4.50 ± 1.25	4.85 ± 1.28	<0.01
Creatinine, Mean ± SD	0.78 ± 0.19	0.77 ± 0.18	0.76 ± 0.21	0.77 ± 0.18	0.81 ± 0.20	<0.01
TG, Mean ± SD	138.43 ± 87.82	92.24 ± 53.85	117.69 ± 64.58	151.54 ± 84.38	192.23 ± 105.10	<0.01
TC, Mean ± SD	198.15 ± 38.93	193.30 ± 38.92	194.93 ± 37.97	199.41 ± 38.47	204.95 ± 39.35	<0.01
HDL, Mean ± SD	50.95 ± 15.35	61.41 ± 17.15	53.41 ± 13.17	47.36 ± 12.21	41.61 ± 10.51	<0.01
CRP, Mean ± SD	2.75 ± 7.68	2.90 ± 11.09	2.35 ± 6.95	2.61 ± 6.01	3.15 ± 5.34	0.14
WC, Mean ± SD	85.68 ± 11.40	73.70 ± 10.22	82.33 ± 5.65	88.77 ± 5.26	97.90 ± 6.50	<0.01
Weight, Mean ± SD	59.44 ± 11.27	51.31 ± 7.73	55.50 ± 7.74	60.68 ± 8.72	70.26 ± 10.62	<0.01
Height, Mean ± SD	1.58 ± 0.08	1.58 ± 0.08	1.57 ± 0.08	1.57 ± 0.08	1.59 ± 0.09	<0.01
BUN, Mean ± SD	15.91 ± 4.53	16.45 ± 4.86	15.82 ± 4.71	15.58 ± 4.34	15.77 ± 4.12	<0.01
LDL, Mean ± SD	119.66 ± 35.38	115.11 ± 33.70	119.26 ± 33.51	121.63 ± 36.06	122.64 ± 37.66	<0.01
Age, Mean ± SD	58.92 ± 8.62	56.98 ± 8.12	58.39 ± 8.25	59.30 ± 8.81	61.01 ± 8.79	<0.01
BMI, Mean ± SD	23.84 ± 3.79	20.57 ± 2.38	22.47 ± 2.21	24.64 ± 2.53	27.70 ± 3.53	<0.01
SBP, Mean ± SD	130.34 ± 20.52	123.70 ± 19.44	126.76 ± 18.59	132.66 ± 20.73	138.24 ± 20.18	<0.01
DBP, Mean ± SD	75.71 ± 11.66	72.60 ± 11.65	74.06 ± 10.73	77.04 ± 11.59	79.16 ± 11.51	<0.01
Gender, n (%)						<0.01
Female	1930 (54.64)	386 (43.71)	526 (59.57)	554 (62.74)	464 (52.55)	
Male	1602 (45.36)	497 (56.29)	357 (40.43)	329 (37.26)	419 (47.45)	
Marital status, n (%)						0.61
Unmarried	518 (14.67)	140 (15.86)	130 (14.72)	128 (14.50)	120 (13.59)	
Married	3014 (85.33)	743 (84.14)	753 (85.28)	755 (85.50)	763 (86.41)	
Smoking status, n (%)						<0.01
never smoker	2211 (62.60)	463 (52.43)	585 (66.25)	610 (69.08)	553 (62.63)	
former smoker	297 (8.41)	52 (5.89)	62 (7.02)	80 (9.06)	103 (11.66)	
current smoker	1024 (28.99)	368 (41.68)	236 (26.73)	193 (21.86)	227 (25.71)	
Drinking, n (%)						<0.01
never drinker	2134 (60.42)	488 (55.27)	543 (61.49)	584 (66.14)	519 (58.78)	
former drinker	296 (8.38)	60 (6.80)	70 (7.93)	74 (8.38)	92 (10.42)	
current drinker	1102 (31.20)	335 (37.94)	270 (30.58)	225 (25.48)	272 (30.80)	
Education, n (%)						0.10
elementary school or below	2515 (71.21)	627 (71.01)	645 (73.05)	627 (71.01)	616 (69.76)	
middle school	926 (26.22)	242 (27.41)	220 (24.92)	229 (25.93)	235 (26.61)	
college or above	91 (2.58)	14 (1.59)	18 (2.04)	27 (3.06)	32 (3.62)	
Rural, n (%)						<0.01
No	566 (16.02)	82 (9.29)	114 (12.91)	171 (19.37)	199 (22.54)	
Yes	2966 (83.98)	801 (90.71)	769 (87.09)	712 (80.63)	684 (77.46)	
Hypertension, n (%)						<0.01
No	2041 (57.79)	658 (74.52)	582 (65.91)	482 (54.59)	319 (36.13)	

Yes	1491 (42.21)	225 (25.48)	301 (34.09)	401 (45.41)	564 (63.87)	
Dyslipidemia, n (%)						<0.01
No	3208 (90.83)	851 (96.38)	828 (93.77)	808 (91.51)	721 (81.65)	
Yes	324 (9.17)	32 (3.62)	55 (6.23)	75 (8.49)	162 (18.35)	
Kidney Disease, n (%)						0.91
No	3370 (95.41)	843 (95.47)	840 (95.13)	841 (95.24)	846 (95.81)	
Yes	162 (4.59)	40 (4.53)	43 (4.87)	42 (4.76)	37 (4.19)	
Liver Disease, n (%)						0.11
No	3432 (97.17)	851 (96.38)	864 (97.85)	864 (97.85)	853 (96.60)	
Yes	100 (2.83)	32 (3.62)	19 (2.15)	19 (2.15)	30 (3.40)	
Antidiabetic drugs, n (%)						<0.01
No	3353 (94.93)	861 (97.51)	853 (96.60)	839 (95.02)	800 (90.60)	
Yes	179 (5.07)	22 (2.49)	30 (3.40)	44 (4.98)	83 (9.40)	
Lipid-lowering agents, n (%)						<0.01
No	3370 (95.41)	868 (98.30)	863 (97.73)	841 (95.24)	798 (90.37)	
Yes	162 (4.59)	15 (1.70)	20 (2.27)	42 (4.76)	85 (9.63)	
Antihypertensive drugs, n (%)						<0.01
No	2867 (81.17)	816 (92.41)	788 (89.24)	704 (79.73)	559 (63.31)	
Yes	665 (18.83)	67 (7.59)	95 (10.76)	179 (20.27)	324 (36.69)	

Table S2 The baseline characteristics stratified by quartiles of eGDR

Variables	Total (n = 3532)	Q1 (n = 883)	Q2 (n = 882)	Q3 (n = 884)	Q4 (n = 883)	P
HbA1c, Mean ± SD	5.41 ± 0.91	5.77 ± 1.17	5.46 ± 1.11	5.33 ± 0.50	5.11 ± 0.46	<.001
FBG, Mean ± SD	120.89 ± 38.66	130.77 ± 50.42	125.10 ± 48.62	114.78 ± 22.37	112.94 ± 19.04	<.001
UA, Mean ± SD	4.46 ± 1.25	4.73 ± 1.32	4.44 ± 1.22	4.47 ± 1.23	4.19 ± 1.16	<.001
Creatinine, Mean ± SD	0.78 ± 0.19	0.80 ± 0.20	0.78 ± 0.21	0.78 ± 0.18	0.76 ± 0.17	<.001
TG, Mean ± SD	138.43 ± 87.82	165.26 ± 95.12	139.88 ± 90.90	136.07 ± 86.36	112.50 ± 68.60	<.001
TC, Mean ± SD	198.15 ± 38.93	205.04 ± 39.27	197.60 ± 36.84	197.65 ± 39.26	192.30 ± 39.30	<.001
HDL, Mean ± SD	50.95 ± 15.35	46.37 ± 13.23	51.36 ± 16.48	50.28 ± 13.63	55.78 ± 16.30	<.001
CRP, Mean ± SD	2.75 ± 7.68	3.56 ± 7.37	2.38 ± 5.65	2.29 ± 5.57	2.78 ± 10.87	0.002
WC, Mean ± SD	85.68 ± 11.40	95.41 ± 7.03	85.35 ± 10.40	87.72 ± 4.87	74.21 ± 10.39	<.001
Weight, Mean ± SD	59.44 ± 11.27	66.71 ± 10.87	58.74 ± 11.55	60.81 ± 8.37	51.49 ± 8.29	<.001
Height, Mean ± SD	1.58 ± 0.08	1.58 ± 0.09	1.58 ± 0.09	1.59 ± 0.08	1.57 ± 0.08	<.001
BUN, Mean ± SD	15.91 ± 4.53	15.77 ± 4.18	16.01 ± 4.91	15.77 ± 4.54	16.08 ± 4.44	0.353
LDL, Mean ± SD	119.66 ± 35.38	125.14 ± 36.74	117.81 ± 34.43	120.36 ± 35.37	115.32 ± 34.23	<.001
Age, Mean ± SD	58.92 ± 8.62	60.54 ± 8.71	60.12 ± 9.04	56.97 ± 7.95	58.05 ± 8.26	<.001
BMI, Mean ± SD	23.84 ± 3.79	26.72 ± 3.48	23.51 ± 3.74	24.17 ± 2.62	20.98 ± 2.79	<.001
SBP, Mean ± SD	130.34 ± 20.52	146.53 ± 19.07	138.56 ± 20.14	118.87 ± 11.50	117.41 ± 12.45	<.001
DBP, Mean ± SD	75.71 ± 11.66	83.03 ± 11.49	79.36 ± 11.43	71.00 ± 8.63	69.47 ± 8.86	<.001
Gender, n (%)						0.154
Female	1930 (54.64)	509 (57.64)	469 (53.17)	486 (54.98)	466 (52.77)	
Male	1602 (45.36)	374 (42.36)	413 (46.83)	398 (45.02)	417 (47.23)	
Marital status, n (%)						0.034
Unmarried	518 (14.67)	122 (13.82)	146 (16.55)	108 (12.22)	142 (16.08)	
Married	3014 (85.33)	761 (86.18)	736 (83.45)	776 (87.78)	741 (83.92)	
Smoking status, n (%)						<.001
never smoker	2211 (62.60)	588 (66.59)	528 (59.86)	567 (64.14)	528 (59.80)	
former smoker	297 (8.41)	90 (10.19)	73 (8.28)	87 (9.84)	47 (5.32)	
current smoker	1024 (28.99)	205 (23.22)	281 (31.86)	230 (26.02)	308 (34.88)	
Drinking, n (%)						<.001
never drinker	2134 (60.42)	539 (61.04)	501 (56.80)	545 (61.65)	549 (62.17)	
former drinker	296 (8.38)	96 (10.87)	88 (9.98)	56 (6.33)	56 (6.34)	
current drinker	1102 (31.20)	248 (28.09)	293 (33.22)	283 (32.01)	278 (31.48)	
Education, n (%)						0.029
elementary school or below	2515 (71.21)	632 (71.57)	645 (73.13)	592 (66.97)	646 (73.16)	
middle school	926 (26.22)	222 (25.14)	216 (24.49)	269 (30.43)	219 (24.80)	
college or above	91 (2.58)	29 (3.28)	21 (2.38)	23 (2.60)	18 (2.04)	
Rural, n (%)						<.001
No	566 (16.02)	183 (20.72)	132 (14.97)	154 (17.42)	97 (10.99)	
Yes	2966 (83.98)	700 (79.28)	750 (85.03)	730 (82.58)	786 (89.01)	
Hypertension, n (%)						<.001
No	2041 (57.79)	5 (0.57)	277 (31.41)	883 (99.89)	876 (99.21)	

Yes	1491 (42.21)	878 (99.43)	605 (68.59)	1 (0.11)	7 (0.79)	
Dyslipidemia, n (%)						<.001
No	3208 (90.83)	714 (80.86)	814 (92.29)	833 (94.23)	847 (95.92)	
Yes	324 (9.17)	169 (19.14)	68 (7.71)	51 (5.77)	36 (4.08)	
Kidney Disease, n (%)						0.999
No	3370 (95.41)	843 (95.47)	841 (95.35)	844 (95.48)	842 (95.36)	
Yes	162 (4.59)	40 (4.53)	41 (4.65)	40 (4.52)	41 (4.64)	
Liver Disease, n (%)						0.970
No	3432 (97.17)	860 (97.40)	856 (97.05)	858 (97.06)	858 (97.17)	
Yes	100 (2.83)	23 (2.60)	26 (2.95)	26 (2.94)	25 (2.83)	
Antidiabetic drugs, n (%)						<.001
No	3353 (94.93)	789 (89.35)	828 (93.88)	864 (97.74)	872 (98.75)	
Yes	179 (5.07)	94 (10.65)	54 (6.12)	20 (2.26)	11 (1.25)	
Lipid-lowering agents, n (%)						<.001
No	3370 (95.41)	792 (89.69)	846 (95.92)	862 (97.51)	870 (98.53)	
Yes	162 (4.59)	91 (10.31)	36 (4.08)	22 (2.49)	13 (1.47)	
Antihypertensive drugs, n (%)						<.001
No	2867 (81.17)	427 (48.36)	675 (76.53)	884 (100.00)	881 (99.77)	
Yes	665 (18.83)	456 (51.64)	207 (23.47)	0 (0.00)	2 (0.23)	

Table S3 Associations of ten insulin resistance surrogate indexes with new-onset stroke or heart disease incidence in patients with prediabetes or diabetes

Stroke	Model I OR (95%CI)	*P*-value	Model II OR (95%CI)	*P*-value	Model III OR (95%CI)	*P*-value	Model IV OR (95%CI)	*P*-value
eGDR (per 1 SD)	0.564(0.431-0.732)	<0.001	0.565(0.430-0.737)	<0.001	0.558(0.316-0.991)	0.0463	0.534(0.030-0.956)	0.03432
eGDR quartile								
Q1	1(Reference)		1(Reference)		1(Reference)		1(Reference)	
Q2	0.771(0.428-1.369)	0.3768	0.740(0.410-1.319)	0.3108	0.634(0.303-1.269)	0.2085	0.583(0.273-1.186)	0.1466
Q3	0.252(0.100-0.551)	0.0012	0.263(0.104-0.581)	0.0019	0.148(0.039-0.539)	0.0039	0.141(0.036-0.525)	0.0037
Q4	0.143(0.042-0.368)	0.0003	0.142(0.041-0.367)	0.0003	0.085(0.0187-0.342)	0.0007	0.079(0.017-0.328)	0.00068
P for trend		<0.001		<0.001		0.0014		0.00105
Heart disease								
eGDR (per 1 SD)	0.687(0.612-0.770)	<0.001	0.716(0.637-0.804)	<0.001	0.807(0.628-1.033)	0.0914	0.795(0.616-1.021)	0.0748
eGDR quartile								
Q1	1(Reference)		1(Reference)		1(Reference)		1(Reference)	
Q2	0.755(0.566-1.005)	0.0555	0.774(0.579-1.033)	0.0833	0.962(0.680-1.354)	0.8279	0.949(0.666-1.344)	0.7721
Q3	0.442(0.316-0.611)	<0.001	0.499(0.355-0.693)	<0.001	0.730(0.401-1.342)	0.3061	0.699(0.381-1.295)	0.2506
Q4	0.411(0.291-0.572)	<0.001	0.452(0.320-0.631)	<0.001	0.668(0.365-1.234)	0.1938	0.651(0.352-1.210)	0.1712
P for trend		<0.001		<0.001		0.2295		0.2104
Stroke								
CVAI (per 1 SD)	1.530(1.192-1.959)	0.0008	1.476(1.160-1.874)	0.0014	1.330(1.016-1.736)	0.0366	1.337(1.017-1.755)	0.0365
CVAI quartile								
Q1	1(Reference)		1(Reference)		1(Reference)		1(Reference)	
Q2	0.898(0.355-2.241)	0.8177	0.999(0.393-2.504)	0.9988	0.901(0.339-2.322)	0.8295	0.945(0.353-2.448)	0.9072
Q3	1.620(0.741-3.716)	0.2344	1.829(0.828-4.233)	0.1419	1.641(0.715-3.909)	0.2475	1.656(0.716-3.978)	0.2433
Q4	2.448(1.197-5.392)	0.0183	2.479(1.202-5.498)	0.0174	1.824(0.830-4.256)	0.1455	1.898(0.852-4.482)	0.1265
P for trend		0.0045		0.0049		0.0739		0.0696
Heart disease								
CVAI (per 1 SD)	1.348(1.205-1.509)	<0.001	1.279(1.137-1.438)	<0.001	1.086(0.950-1.240)	0.2242	1.088(0.948-1.248)	0.2262
CVAI quartile								

Q1	1(Reference)		1(Reference)		1(Reference)		1(Reference)	
Q2	1.107(0.770-1.593)	0.5837	0.993(0.688-1.435)	0.9719	1.019(0.696-1.498)	0.9199	0.997(0.678-1.471)	0.9894
Q3	1.492(1.060-2.111)	0.0227	1.279(0.903-1.822)	0.169	1.047(0.717-1.534)	0.813	1.004(0.682-1.483)	0.9839
Q4	2.065(1.495-2.881)	<0.001	1.732(1.242-2.435)	0.0014	1.174(0.806-1.722)	0.4047	1.149(0.781-1.701)	0.4816
P for trend		<0.001		0.0002		0.3774		0.4557

Model I was unadjusted; Model II included adjustments for gender and age; and Model III contained additional adjustments for marital status, smoking status, drinking status, education level, residence, hypertension, dyslipidemia, kidney disease, liver disease, antidiabetic drugs, lipid-lowering agents, antihypertensive drugs. Model IV further adjusted for TC, LDL, BUN, Creatinine, SBP, and DBP.

Table S4 Associations of ten insulin resistance surrogate indexes with new-onset cardiovascular disease incidence in patients with prediabetes or diabetes after excluding participants with incomplete covariate data

	Model I OR (95%CI)	P-value	Model II OR (95%CI)	P-value	Model III OR (95%CI)	P-value	Model IV OR (95%CI)	P-value
eGDR (per 1 SD)	0.687(0.635-0.744)	<0.001	0.711(0.656-0.770)	<0.001	0.799(0.676-0.944)	0.0087	0.816(0.689-0.965)	0.018
eGDR quartile								
Q1	1 (Reference)		1 (Reference)		1 (Reference)		1 (Reference)	
Q2	0.609(0.495-0.749)	<0.001	0.625(0.507-0.770)	<0.001	0.730(0.570-0.933)	0.0124	0.741(0.578-0.949)	0.0178
Q3	0.381(0.304-0.476)	<0.001	0.421(0.335-0.529)	<0.001	0.468(0.314-0.699)	0.0002	0.486(0.325-0.728)	0.0005
Q4	0.392(0.313-0.489)	<0.001	0.423(0.337-0.529)	<0.001	0.481(0.322-0.719)	0.0003	0.505(0.337-0.758)	0.0009
P for trend		<0.001		<0.001		0.0018		0.0042
CVAI (per 1 SD)								
CVAI quartile	1.387(1.283-1.502)	<0.001	1.327(1.224-1.439)	<0.001	1.146(1.049-1.252)	0.0026	1.130(1.032-1.237)	0.008
Q1	1 (Reference)		1 (Reference)		1 (Reference)		1 (Reference)	
Q2	1.292(1.020-1.639)	0.0341	1.190(0.936-1.514)	0.1557	1.131(0.886-1.445)	0.3226	1.108(0.867-1.417)	0.4138
Q3	1.435(1.136-1.816)	0.0025	1.272(1.003-1.617)	0.0476	1.083(0.846-1.388)	0.5272	1.039(0.809-1.335)	0.7638
Q4	2.322(1.858-2.911)	<0.001	2.021(1.609-2.545)	<0.001	1.392(1.086-1.785)	0.0091	1.331(1.035-1.714)	0.0263
P for trend		<0.001		<0.001		0.0159		0.045

Model I was unadjusted; Model II included adjustments for gender and age; and Model III contained additional adjustments for marital status, smoking status, drinking status, education level, residence, hypertension, dyslipidemia, kidney disease, liver disease, antidiabetic drugs, lipid-lowering agents, antihypertensive drugs. Model IV further adjusted for TC, LDL, BUN, Creatinine, SBP, and DBP.

Table S5 Associations of ten insulin resistance surrogate indexes with new-onset cardiovascular disease incidence in patients with prediabetes or diabetes after excluding participants who developed CVD in wave 2 during follow-up

	Model I OR (95%CI)	P-value	Model II OR (95%CI)	P-value	Model III OR (95%CI)	P-value	Model IV OR (95%CI)	P-value
eGDR (per 1 SD)	0.688(0.633-0.746)	<0.001	0.709(0.653-0.770)	<0.001	0.803(0.673-0.956)	0.0145	0.824(0.690-0.984)	0.0333
eGDR quartile								
Q1	1(Reference)		1(Reference)		1(Reference)		1(Reference)	
Q2	0.609(0.491-0.753)	<0.001	0.623(0.502-0.772)	<0.001	0.705(0.543-0.913)	0.0083	0.725(0.557-0.941)	0.0162
Q3	0.393(0.312-0.495)	<0.001	0.429(0.339-0.542)	<0.001	0.475(0.312-0.723)	0.0005	0.496(0.325-0.75)	0.0011
Q4	0.392(0.310-0.493)	<0.001	0.419(0.332-0.529)	<0.001	0.476(0.313-0.725)	0.0005	0.508(0.333-0.778)	0.0018
P for trend		<0.001		<0.001		0.0021		0.0062
CVAI (per 1 SD)								
CVAI quartile	1.389(1.281-1.508)	<0.001	1.335(1.228-1.452)	<0.001	1.156(1.053-1.268)	0.0023	1.136(1.033-1.249)	0.0086
Q1	1(Reference)		1(Reference)		1(Reference)		1(Reference)	
Q2	1.394(1.089-1.787)	0.0084	1.293(1.008-1.663)	0.0437	1.207(0.934-1.562)	0.1523	1.175(0.907-1.524)	0.2233
Q3	1.394(1.089-1.788)	0.0084	1.250(0.973-1.609)	0.0819	1.074(0.825-1.399)	0.5957	1.029(0.788-1.346)	0.8325
Q4	2.423(1.921-3.067)	<0.001	2.137(1.686-2.718)	<0.001	1.460(1.123-1.901)	0.0048	1.373(1.051-1.797)	0.0204
P for trend		<0.001		<0.001		0.0144		0.05

Model I was unadjusted; Model II included adjustments for gender and age; and Model III contained additional adjustments for marital status, smoking status, drinking status, education level, residence, hypertension, dyslipidemia, kidney disease, liver disease, antidiabetic drugs, lipid-lowering agents, antihypertensive drugs. Model IV further adjusted for TC, LDL, BUN, Creatinine, SBP, and DBP.

Table S6 Associations of ten insulin resistance surrogate indexes with new-onset cardiovascular disease incidence in patients with prediabetes or diabetes after excluding participants receiving antihyperglycemic, antihypertensive, or lipid-lowering treatments at baseline

	Model I OR (95%CI)	P-value	Model II OR (95%CI)	P-value	Model III OR (95%CI)	P-value	Model IV OR (95%CI)	P-value
eGDR (per 1 SD)	0.834(0.757-0.918)	0.00023	0.858(0.778-0.945)	0.00202	0.824(0.0.680-0.997)	0.0479	0.826(0.682-0.997)	0.0488
eGDR quartile								
Q1	1(Reference)		1(Reference)		1(Reference)		1(Reference)	
Q2	0.765(0.585-0.999)	0.0498	0.816(0.622-1.068)	0.1401	0.662(0.408-1.050)	0.0863	0.680(0.418-1.081)	0.11
Q3	0.648(0.491-0.852)	0.0019	0.710(0.536-0.937)	0.0162	0.539(0.311-0.921)	0.0255	0.567(0.326-0.972)	0.0413
Q4	0.339(0.484-0.841)	0.0015	0.676(0.512-0.893)	0.0059	0.522(0.301-0.893)	0.0189	0.556(0.319-0.956)	0.0357
P for trend		0.00059		0.00329		0.0464		0.0708
CVAI (per 1 SD)								
CVAI quartile	1.230(1.116-1.356)	<0.001	1.181(1.068-1.305)	0.00115	1.167(1.052-1.296)	0.0035	1.138(1.022-1.267)	0.0181
Q1	1(Reference)		1(Reference)		1(Reference)		1(Reference)	
Q2	1.438(1.074-1.931)	0.015	1.336(0.995-1.800)	0.0552	1.322(0.981-1.789)	0.0681	1.300(0.962-1.764)	0.085
Q3	1.357(1.011-1.826)	0.0427	1.225(0.908-1.658)	0.1847	1.222(0.900-1.664)	0.1995	1.194(0.877-1.631)	0.2614
Q4	1.778(1338-2.369)	<0.001	1.561(1.167-2.093)	0.00276	1.497(1.108-2.029)	0.0089	1.441(1.061-1.966)	0.0201
P for trend		0.00029		0.00812		0.0221		0.0461

Model I was unadjusted; Model II included adjustments for gender and age; and Model III contained additional adjustments for marital status, smoking status, drinking status, education level, residence, hypertension, dyslipidemia, kidney disease, liver disease, antidiabetic drugs, lipid-lowering agents, antihypertensive drugs. Model IV further adjusted for TC, LDL, BUN, Creatinine, SBP, and DBP.

Table S7 Feature variables selected by the two feature selection methods

Variables	LASSO	Boruta algorithm
HbA1c		√
FBG		
UA	√	√
Creatinine		
TG	√	
TC		
HDL	√	
CRP		
WC	√	√
Weight		
Height		
BUN	√	√
LDL	√	
Gender	√	√
Age	√	√
BMI	√	√
SBP	√	
DBP	√	√
Marital status	√	
Smoking status		
Drinking		
Education level	√	√
Rural		
Hypertension	√	√
Dyslipidemia	√	√
Kidney disease		√
Liver disease		√
Antidiabetic drugs		
Lipid-lowering agents		
Antihypertensive drugs		

Table S8 Performance comparison of nine machine learning models incorporating eGDR and CVAI for predicting CVD incidence

Model	Accuracy	Precision	Recall	F1	Sensitivity	Specificity
KNN	0.8188	0.577279	1	0.731993	1	0.759217
LightGBM	0.765289	0.520071	0.667048	0.584461	0.667048	0.797592
CatBoost	0.712344	0.435219	0.545767	0.484264	0.545767	0.767118
GBM	0.698754	0.425197	0.617849	0.503731	0.617849	0.725357
SVM	0.698471	0.366806	0.300915	0.33061	0.300915	0.829195
Xgboost	0.677803	0.388514	0.526316	0.447036	0.526316	0.727615
Logistic	0.6141	0.350824	0.657895	0.45762	0.657895	0.599699
NeuralNetwork	0.60504	0.344848	0.662471	0.453584	0.662471	0.586155
Adaboost	0.531993	0.303382	0.687643	0.421016	0.687643	0.480813
